# Optimizations of SiRNA Design for the Activation of Gene Transcription by Targeting the TATA-Box Motif

**DOI:** 10.1371/journal.pone.0108253

**Published:** 2014-09-24

**Authors:** Miaomiao Fan, Yijun Zhang, Zhuoqiong Huang, Jun Liu, Xuemin Guo, Hui Zhang, Haihua Luo

**Affiliations:** 1 Institute of Human Virology, Zhongshan School of Medicine, Sun Yat-sen University, Guangzhou, Guangdong, China; 2 Key Laboratory of Tropical Disease Control of Ministry of Education, Zhongshan School of Medicine, Sun Yat-sen University, Guangzhou, Guangdong, China; Institute of Genetics and Molecular and Cellular Biology, France

## Abstract

Small interfering RNAs (siRNAs) are widely used to repress gene expression by targeting mRNAs. Some reports reveal that siRNAs can also activate or inhibit gene expression through targeting the gene promoters. Our group has found that microRNAs (miRNAs) could activate gene transcription via interaction with the TATA-box motif in gene promoters. To investigate whether siRNA targeting the same region could upregulate the promoter activity, we test the activating efficiency of siRNAs targeting the TATA-box motif of 16 genes and perform a systematic analysis to identify the common features of the functional siRNAs for effective activation of gene promoters. Further, we try various modifications to improve the activating efficiency of siRNAs and find that it is quite useful to design the promoter-targeting activating siRNA by following several rules such as (a) complementary to the TATA-box-centered region; (b) UA usage at the first two bases of the antisense strand; (c) twenty-three nucleotides (nts) in length; (d) 2′-O-Methyl (2′-OMe) modification at the 3′ terminus of the antisense strand; (e) avoiding mismatches at the 3′ end of the antisense strand. The optimized activating siRNAs potently enhance the expression of *interleukin-2* (*IL-2*) gene in human and mouse primary CD4^+^ T cells with a long-time effect. Taken together, our study provides a guideline for rational design the promoter-targeting siRNA to sequence-specifically enhance gene expression.

## Introduction

SiRNAs are widely used to repress genes expression by targeting mRNAs [1]–[6]. However, so far, there is not an easy-to-use method to upregulate gene expression specifically. It has been reported that cellular miRNAs and synthetic siRNAs could inhibit or induce gene expression through targeting gene promoters. MiR-373 can induce the expression of E-cadherin and cold-shock domain-containing protein C2 (CSDC2) by targeting their promoters [7]. MiR-423-5p causes transcriptional silencing of progesterone receptor (PR) by targeting a highly conserved region in the promoter [8]. It was reported that a short hairpin RNA (shRNA), shPromA, induced highly specific transcriptional gene silencing of HIV-1 through targeting the NFκB binding sequences of the HIV-1 promoter [9], [10]. MiR-744 and miR-1186 induce Ccnb1 expression and manipulate mouse cell proliferation with putative binding site in the gene promoter [11]. In addition, double-stranded RNAs (dsRNAs) targeting promoter regions of E-cadherin, p21^WAF1/CIP1^ (p21) or VEGF showed long-lasting and sequence-specific induction of targeted genes [12]. A research group has shown that promoter-directed antigene RNAs (agRNAs) could activate or silence gene expression and Argonaute2 (AGO2) was involved in the process [13]–[15]. However, previous studies have not yet identified any unique feature of the binding site(s) for these activating small RNAs. Also, it is difficult to predict whether a siRNA targeting a sequence in the promoter will have either up- or down- regulatory effects.

Recently, our group revealed that an HIV-1-encoded miRNA, miR-H3, could target the TATA-box region in HIV-1 5′ LTR and enhance viral replication. Moreover, the chemically-synthesized siRNAs targeting the same site could activate HIV-1 viral production from the CD4^+^ T cells isolated from HIV-1-infected patients receiving suppressive highly active antiretroviral therapy (HAART) [16]. We then extended these findings to the cellular miRNAs and demonstrated that a certain amount of cellular miRNAs were associated with the RNA polymerase II (Pol II) core transcription machinery and able to activate gene transcription via interaction with the TATA-box motif (see the related manuscript). Since TATA-box motif is within a narrow range at immediate upstream of the transcription start site (TSS) and is easy to be identified, it is intriguing to evaluate the potential of activating gene expression with the siRNAs targeting the TATA-box region and identify the features that contribute to the functionality of these activating siRNAs.

For rational design of the conventional siRNAs to degrade target mRNAs, many guidelines have been developed. For instance, the G/C content of the siRNA sense strand should be 30–50%; bases at positions 1–3 should be A/U; the 10th and 19th bases of the sense strand should be A or U; the 1st base of the sense strand should be G/C [17]–[20]. Traditional 21 nts siRNA duplexes contain 19 base pairs, with 2 nts overhangs toward their 3′ termini [21]–[23]. Besides, the two nucleotides at 3′- overhang are critical for siRNA function and the most efficient siRNAs contained UG, UU, TdG or TT at the 3′- overhangs [24], [25]. Moreover, it has been demonstrated that the synthetic 19 nts siRNAs mediate gene inhibition efficiently in the cytoplasm *in vitro* [1]–[5] or *in vivo* [6]. However, it has been reported that the synthetic 25–30 nts RNA duplexes were more potent than their 19 nts long counterparts, especially the 21-mer siRNAs with no overhang [26]. Zamore and colleagues noted that siRNA duplexes were functionally asymmetric [27]. The strand with less stable 5′ end starting with an A-U pair was incorporated into the RNA-induced silencing complex (RISC) more efficiently. Interestingly, miRNAs or tRNAs with specific 3′ sequences (ASUS (S = C or G) motif containing sequences: AGUGUU, ACUGUU, AGUGAU, and so on) could accumulate in the nucleus [28]–[30] and an Argonaute-like protein NRDE-3 could transport siRNAs from the cytoplasm to the nucleus in *Caenorhabditis elegans* [31].

A large amount of chemical modifications have been developed to improve the properties of siRNAs [32]–[34]. These modifications could be on phosphodiester backbone, base or ribose. Therein, since ribose 2′-OH was not required for siRNA function [35], lots of 2′-modifications have been introduced, such as 2′-O-Methyl (2′-OMe), 2′-fluoro (2′-F), 2′-O-methoxyethyl (2′-O-MOE), 2′-aminoethyl (2′-AE), 2′-guanidinopropyl (2′-GP) and locked nucleic acid (LNA) [36]. They can be used alone or in combination, but some cannot be placed at certain positions [34], [37]. The 2′-OMe which naturally occurs is a commonly used 2′- modification [37]–[40]. In addition, some chemical modifications can reduce off-target effects and the recipient immune responses induced by siRNAs [41]–[43], make siRNAs more resistant to metabolic degradation [41], [44], [45] and improve their pharmacokinetic properties [46], [47]. Moreover, when bearing a 5′ phosphate, single stranded siRNAs (ss-siRNAs) also function in the RNA interfering (RNAi) pathway, though their potency is significantly lower than siRNA duplexes [48]. Recently, several studies showed that chemical modifications dramatically improved activities of ss-siRNAs [49]–[51].

Although several criteria have been suggested for efficient design of repressive siRNAs that target mRNAs, there is no suggestion about the effective design of activating siRNAs that target gene promoters. In this study, we attempted to investigate the role of TATA-box-targeting siRNAs in the regulation of gene expression and increase their efficiency by testing various modifications including the target site, sequence characters, length and chemical modifications. We identified several helpful characteristics and have combined them together to obtain a reasonable strategy for rational designing siRNAs to activate the gene promoters specifically.

## Materials and Methods

### Ethics statement

This research was approved by the Ethics Review Board of Sun Yat-Sen University. Healthy donors were comprised of a group of local volunteers, who were seronegative and had no reported history of chronic illness or intravenous drug use. All mouse experiments were approved by the Sun Yat-Sen University Institutional Animal Care and Use Committee. BALB/c mice were obtained from the Animal Experimental Center of Sun Yat-Sen University. All mice were maintained under specific pathogen-free conditions and used at 8–10 weeks of age.

### Promoter sequence, TSS position, TATA box and the sequences of the two mutated *IL-2* promoter

The sequences of gene promoters were downloaded from the Eukaryotic Promoter Database (EPD, http://epd.vital-it.ch/) which is a collection of experimentally defined eukaryotic POL II promoters [52]. The TSS positions provided by EPD are inferred from next-generation sequencing data including mRNA 5′ tags and chromatin signatures. Besides, the position of each promoter sequence was confirmed in the UCSC genome browser with the gene transcription data. The TATA-box motif of each gene is validated from EPD. The sequence of TATA-box motif was also confirmed by using the program YAPP Eukaryotic Core Promoter Predictor (http://www.bioinformatics.org/yapp/cgi-bin/yapp.cgi). The sequences of the two mutated *IL-2* promoter (−50 to +1) were as follows: IL-2mt-27/−25, TAATATTTTTCCAGAATTAACAG*C*A*C*AAATTGCATCTCTTGTTCAAGAGTTC; IL-2mt-17/−16, TAATATTTTTCCAGAATTAACAGTATAAATTGC*TA*CTCTTGTTCAAGAGTTC. Mutations were italicized and underlined.

### Plasmids, miRNA mimics, siRNAs and single-strand RNAs (ssRNAs)

The *IL-2* promoter-driven luciferase reporter vector was constructed by replacing the CMV promoter of pMIR-REPORT vector (Applied Biosystems) with the sequence −400 to +1 bp relative to the TSS of the human *IL-2* promoter. The vectors containing HIV-1, *apolipoprotein E* (*APOE*), *insulin* (*INS*), *c-Myc*, or other gene promoters were generated in the same way. Hsa-let-7i mimics, ssRNAs and the corresponding negative control RNAs were purchased from Genepharma (Shanghai, China). SiRNAs targeting the TATA-box motif of each gene were synthesized and chemically modified by Ribobio (Guangzhou, China). The sequences of the small RNAs used in this study are listed in Table 1–3 and Table S1–S3 in File S1.

**Table 1:** Sequences of synthesized oligonucleotides targeting human *IL-2*, *insulin* (*INS*) or mouse *IL-2* promoter.

Gene	siRNAs	Sense strand (5′–3′)
Human *IL-2*	IL-2-CEN	AACAG**UAUAAAUU**GCAUCU-*dTdT*
	IL-2-CEN−6	AGAAUUAACAG**UAUAAAUU-** *dTdT*
	IL-2-CEN−4	AAUUAACAG**UAUAAAUU**GC-*dTdT*
	IL-2-CEN−2	UUAACAG**UAUAAAUU**GCAU-*dTdT*
	IL-2-CEN+2	CAG**UAUAAAUU**GCAUCUCU-*dTdT*
	IL-2-CEN+4	G**UAUAAAUU**GCAUCUCUUG-*dTdT*
	IL-2-CEN+6	**AUAAAUU**GCAUCUCUUGUU-*dTdT*
	IL-2-5u	AACAG**UAUAAAUU**GCAUC**a-** *dTdT*
	IL-2-5ua (5ua-19)	AACAG**UAUAAAUU**GCAU**ua**-*dTdT*
	IL-2-5ua-15	CAG**UAUAAAUU**GCAU**-** *dTdT*
	IL-2-5ua-17	ACAG**UAUAAAUU**GCAU**a**-*dTdT*
	IL-2-5ua-21	**c**UAACAG**UAUAAAUU**GCAU**ua**-*dTdT*
	IL-2-5ua-23	**c**AUUAACAG**UAUAAAUU**GCAU**ua**-*dTdT*
	IL-2-5ua-25	**c**AUUAACAG**UAUAAAUU**GCAUCU**ua**-*dTdT*
	IL-2-5ua-27	GAAUUAACAG**UAUAAAUU**GCAUCUCU**a**-*dTdT*
	IL-2-5ua-29	CAGAAUUAACAG**UAUAAAUU**GCAUCUCU**a**-*dTdT*
Mouse *IL-2*	ms-IL-2-CEN	AACAG**UAUAAAUU**GCCTCC-*dTdT*
	ms-IL-2-5ua21	**c**UAACAG**UAUAAAUU**GCCU**ua**-*dTdT*
	ms- IL-2-5ua23	CAUUAACAG**UAUAAAUU**GCCU**ua**-*dTdT*
Human *INS*	INS-CEN	UGAGAC**UAUAAAGC**CAGCG-*dTdT*
	INS-CEN−6	GGGCUCUGAGAC**UAUAAAG**-*dTdT*
	INS-CEN−4	GCUCUGAGAC**UAUAAAGC**C-*dTdT*
	INS-CEN−2	UCUGAGAC**UAUAAAGC**CAG-*dTdT*
	INS-CEN+2	AGAC**UAUAAAGC**CAGCGGG-*dTdT*
	INS-CEN+4	AC**UAUAAAGC**CAGCGGGGG-*dTdT*
	INS-CEN+6	**UAUAAAGC**CAGCGGGGGCC-*dTdT*
Human *APOE*	APOE-CEN	GAGCCC**UAUAAUUG**GACAA-*dTdT*
	APOE-CEN−6	CAGGGGGAGCCC**UAUAAUU**-*dTdT*
	APOE-CEN−4	GGGGGAGCCC**UAUAAUUG**G-*dTdT*
	APOE-CEN−2	GGGAGCCC**UAUAAUUG**GAC-*dTdT*
	APOE-CEN+2	GCCC**UAUAAUUG**GACAAGT-*dTdT*
	APOE-CEN+4	CC**UAUAAUUG**GACAAGTCT-*dTdT*
	APOE-CEN+6	**UAUAAUUG**GACAAGTCTGG-*dTdT*

For each siRNA, just the sense strand is shown. Nucleotides in bold were corresponding to TATA-box motif. Modified bases were in bold lowercase. ms, mouse.

**Table 2:** Sequence mutations in the siRNA IL2-CEN or INS-CEN.

	si-IL-2		si-INS
wt(CEN)	5′-AACAGUAUAAAUUGCAUCU-*dTdT*-3′	wt(CEN)	5′-UGAGACUAUAAAGCCAGCG-*dTdT*-3′
	3′-*dTdT*-UUGUCAUAUUUAACGUAGA-5′		3′-*dTdT*-ACUCUGAUAUUUCGGUCGC-5′
s2	5′-AACAGUAUAAAUUGCAU**g**U-*dTdT*-3′	s2	5′-UGAGACUAUAAAGCCAG**g**G-*dTdT*-3′
	3′-*dTdT*-UUGUCAUAUUUAACGUA**c**A-5′		3′-*dTdT*-ACUCUGAUAUUUCGGUC**c**C-5′
s5	5′-AACAGUAUAAAUUG**g**AUCU-*dTdT*-3′	s3	5′-UGAGACUAUAAAGCCA**c**CG-*dTdT*-3′
	3′-*dTdT*-UUGUCAUAUUUAAC**c**UAGA-5′		3′-*dTdT*-ACUCUGAUAUUUCGGU**g**GC-5′
s6	5′-AACAGUAUAAAUU**c**CAUCU-*dTdT*-3′	s5	5′-UGAGACUAUAAAGC**g**AGCG-*dTdT*-3′
	3′-*dTdT*-UUGUCAUAUUUAA**g**GUAGA-5′		3′-*dTdT*-ACUCUGAUAUUUCG**c**UCGC-5′
ds2/5	5′-AACAGUAUAAAUUG**g**AU**g**U-*dTdT*-3′	ds2/3	5′-UGAGACUAUAAAGCCA**cg**G-*dTdT*-3′
	3′-*dTdT*-UUGUCAUAUUUAAC**c**UA**c**A-5′		3′-*dTdT*-ACUCUGAUAUUUCGGU**gc**C-5′
ds5/6	5′-AACAGUAUAAAUU**cg**AUCU-*dTdT*-3′	ds3/5	5′-UGAGACUAUAAAGC**g**A**c**CG-*dTdT*-3′
	3′-*dTdT*-UUGUCAUAUUUAA**gc**UAGA-5′		3′-*dTdT*-ACUCUGAUAUUUCG**c**U**g**GC-5′
ds11/12	5′-AACAGUA**au**AAUUGCAUCU-*dTdT*-3′	ds10/11	5′-UGAGACUA**au**AAGCCAGCG-*dTdT*-3′
	3′-*dTdT*-UUGUCAU**ua**UUAACGUAGA-5′		3′-*dTdT*-ACUCUGAU**ua**UUCGGUCGC-5′
s15	5′-AACA**c**UAUAAAUUGCAUCU-*dTdT*-3′	s14	5′-UGAGA**g**UAUAAAGCCAGCG-*dTdT*-3′
	3′-*dTdT*-UUGU**g**AUAUUUAACGUAGA-5′		3′-*dTdT*-ACUCU**c**AUAUUUCGGUCGC-5′
s17	5′-AA**g**AGUAUAAAUUGCAUCU-*dTdT*-3′	s16	5′-UGA**c**ACUAUAAAGCCAGCG-*dTdT*-3′
	3′-*dTdT*-UU**c**UCAUAUUUAACGUAGA-5′		3′-*dTdT*-ACU**g**UGAUAUUUCGGUCGC-5′
ds15/17	5′-AA**g**A**c**UAUAAAUUGCAUCU-*dTdT*-3′	ds14/16	5′-UGA**c**A**g**UAUAAAGCCAGCG-*dTdT*-3′
	3′-*dTdT*-UU**c**U**g**AUAUUUAACGUAGA-5′		3′-*dTdT*-ACU**g**U**c**AUAUUUCGGUCGC-5′

Single (s) and double (ds) mutants were all named according to the position (from the 5′ end of the antisense strand) of the mutation. All mutations (in bold lowercase) were GC inversions relative to wild-type (wt), except that ds11/12 of IL2-CEN and ds10/11 of INS-CEN were mutated in the TATA-box.

**Table 3:** Chemical modifications of siRNAs targeting the *IL-2* TATA-box.

Name	Sequence (n: 2′-OMe, N: 2′-F)
Me3	5′-AACAGUAUAAAUUGCAUCU-*dTdT*-3′
	3′-*dTdT*-U**uguc**AUAUUUAACGUAGA-5
Me5	5′-AACAGUAUAAAUUGCAUCU-*dTdT*-3′
	3′-*dTdT*-UUGUCAUAUUUAACG**uag**A-5′
MeF3	5′-AACAGUAUAAAUUGCAUCU-*dTdT*-3′
	3′-*dTdT*-UU **g** UCAUAUUUAACGUAGA-5′
MeF5	5′-AACAGUAUAAAUUGCAUCU-*dTdT*-3′
	3′-*dTdT*-UUGUCAUAUUUAACGU **ag**A-5′
MeAll	5′-AACAGUAUAAAUUGCAUCU-*dTdT*-3′
	3′-*dTdT*-**uugucauauuuaacguaga**-5′
MeFAlter	5′-AACAGUAUAAAUUGCAUCU-*dTdT*-3′
	3′-*dTdT*-**u** U **g** UC **a** U **a** U **u** U **aa** C **g** U **aga**-5′
5ua21Me3	5′-CUAACAGUAUAAAUUGCAUUA-*dTdT*-3′
	3′-*dTdT*-G**auug**UCAUAUUUAACGUAAU-5′
5ua23Me3	5′-CAUUAACAGUAUAAAUUGCAUUA-*dTdT*-3′
	3′-*dTdT*-G**uaau**UGUCAUAUUUAACGUAAU-5′
ms-5ua23Me3	5′-CAUUAACAGUAUAAAUUGCCUUA-*dTdT*-3′
	3′-*dTdT*-G**uaau**UGUCAUAUUUAACGGAAU-5′

The modified residues in antisense strands of siRNAs targeting human or mouse (ms) *IL-2* promoter were in bold lowercase or underlined. Me, 2′-O-Methyl; F, 2′-fluoro.

### Cell cultures

Jurkat and HEK293T cells were obtained from ATCC (American Type Culture Collection, Manassas, VA) and cultured according to the recommendations. Human peripheral blood mononuclear cells (PBMCs) were isolated from the whole blood of healthy donors with Ficoll-Hypaque Solution (HAO YANG, Tianjin, China). The human primary CD4^+^ T lymphocytes were then isolated from PBMCs with human CD4^+^ T Lymphocyte Enrichment kit (BD). The mouse primary CD4^+^ T cells were isolated from spleens of 8- to 10-week-old female BALB/c mice with mouse CD4^+^ T Lymphocyte Enrichment kit (BD). The isolated human or mouse primary CD4^+^ T cells were grown in RPMI 1640 media supplemented with 10% fetal bovine serum (FBS), 50 U/ml penicillin and 50 µg/ml streptomycin in a humidified incubator with 5% CO_2_.

### Transfection

Small RNAs and plasmids were transfected into HEK293T cells with Lipofectamine 2000 (Invitrogen) according to the manufacturer’s protocol. SiRNAs were transfected into Jurkat and human or mouse primary CD4^+^ T cells with Lipofectamine RNAiMAX (Invitrogen) at final concentrations of 120 nM. At 12 hrs post transfection, Jurkat cells were treated with 50 ng/ml phorbol 12-myristate 13-acetate (PMA, Sigma) and 1 µM ionomycin (Sigma) for 36 or 84 hrs to activate the cells. Human CD4^+^ T cells were activated with 1 µg/ml anti-CD3 (R&D Systems) and 5 µg/ml anti-CD28 (R&D Systems) for 84 hrs. Mouse CD4^+^ T cells were stimulated with 2 µg/ml anti-mouse CD3 (R&D Systems) and 1 µg/ml anti-mouse CD28 (R&D Systems) for 84 hrs.

### Dual-luciferase reporter assay

HEK293T cells were seeded in 48-well plates (Corning) at a density of 2.5×10^4^ cells per well and grown to 40–60% confluence overnight. Five to ten ng of gene promoter-driven firefly luciferase (FL) reporter and 2 ng renilla luciferase (RL) vector were co-transfected with activating siRNAs or negative control siRNA at final concentrations of 25 nM into HEK293T cells using Lipofectamine 2000 (Invitrogen) by following the manufacturer’s instructions. At 36 hrs post transfection, FL and RL activities were measured with the Dual-Glo Luciferase assay system (Promega) according to the instructions of the manufacturer. FL signals (sample) were normalized to RL signals (transfection control).

### Quantitative real-time RT-PCR analysis

Total RNAs from Jurkat or primary CD4^+^ T cells were isolated with TRIzol reagent (Invitrogen) and then subjected to cDNA synthesis using PrimeScript RT reagent Kit (Takara). Quantitative PCR was performed with SYBR Premix ExTaq II Kit (Takara) by using the CFX96 Real-Time System (Bio-Rad). The instructions of the manufacturer were followed. Quantification was normalized to mRNA levels of the housekeeping gene GAPDH or β-actin. The sequences of the primers used in this study are listed in the supplementary data.

### Western blot

After transfection, cells were collected and lysed with lysis buffer [150 mM NaCl, 50 mM Tris-HCl (pH 7.5), 1 mM EDTA, 1% Triton X-100, and 0.5% NP-40]. The lysates were then separated by 15% SDS-PAGE and analyzed by immunoblotting using primary antibodies specific for human IL-2 (rabbit monoclonal, Abcam) or β-actin (mouse monoclonal, BD). The LI-COR Odyssey scanner was used to detect and quantify fluorescent signals as previously described [53].

## Results

### SiRNAs targeting the center of TATA-box region activate gene promoter transcription most efficiently

**Figure 1:**
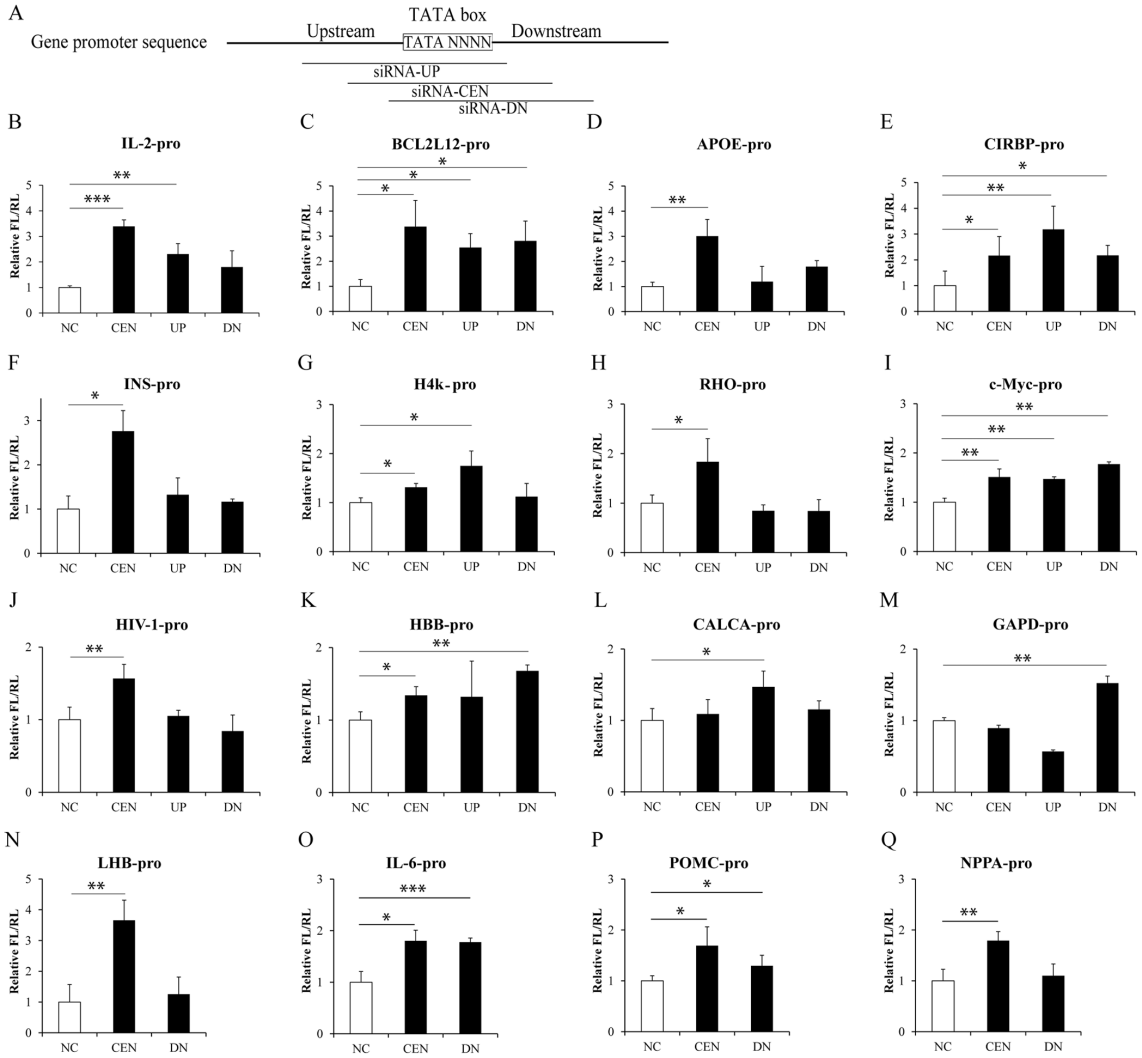
TATA-box motif targeted siRNAs activate the promoter activities of 16 genes. (**A**) Schematic diagram for designing siRNAs that target the TATA-box motif. The consensus sequence of TATA-box motif was highlighted. SiRNAs targeting the TATA-box-centered position (CEN), siRNAs targeting the positions in upstream (UP) or downstream (DN) of the TATA-box were indicated. (**B**–**Q**) Analyses of promoter activities of 16 genes with siRNAs targeting different sites of the TATA-box motif region. The indicated siRNAs were co-transfected with the target promoter-driven firefly luciferase (FL) and renilla luciferase (RL) constructs into HEK293T cells. Thirty-six hrs later, promoter activities were determined by dual-luciferase assay. NC, negative control siRNA. *P*-values were calculated using the two tailed unpaired Student’s t-test with equal variances. n = 3. *, *p*<0.05. **, *p*<0.01. ***, *p*<0.001.

Our previous studies have demonstrated that many cellular miRNAs upregulated gene transcription by targeting the TATA-box motif in gene promoters. For example, miRNA let-7i could upregulate human *IL-2* promoter activity. We wondered whether siRNAs targeting the TATA-box region in gene promoters could also activate their promoter activities. For this purpose, we randomly designed three siRNAs targeting a ∼30 bp range around the individual TATA-box of sixteen genes: siRNAs targeting the TATA-box-centered position (CEN), siRNAs targeting the positions in upstream (UP) or downstream (DN) of the TATA-box in each gene promoter (Fig. 1A). The sequences of these siRNAs were listed in Table S1 in File S1. After these siRNAs or the negative control siRNA (NC) co-transfected respectively with the target promoter-driven firefly luciferase (FL) and CMV promoter-driven renilla luciferase (RL) constructs into HEK293T cells, promoter activities were independently determined by dual-luciferase assay. We found that almost all the siRNAs CEN activated the target gene promoter activity (14 out of 16), although certain siRNA UP or DN was more efficient in some cases (Fig. 1B–Q).

**Figure 2:**
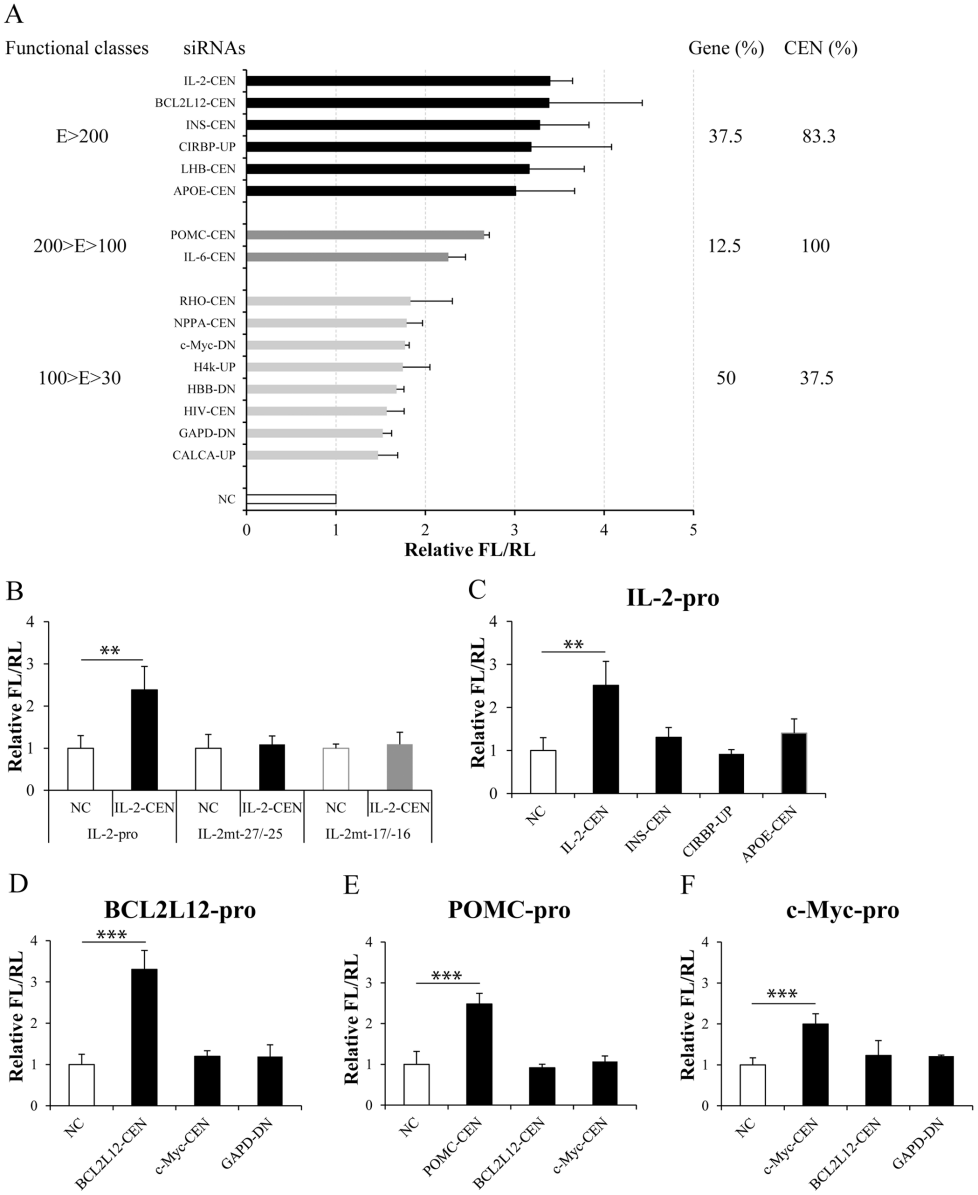
SiRNAs targeting TATA-box enhance the promoter activities specifically and efficiently. (**A**) Effects of siRNAs targeting TATA-box motif on promoter activities of 16 genes in Figure 1. The most effective siRNA for each gene was listed in the y-axis, and the promoter activities relative to NC were listed in the x-axis. These genes and functional activating siRNAs were classified according to the enhancement of siRNAs on promoter activities compared with NC (E (%)) as shown in Table S1 in File S1. Promoter activities were determined with dual-luciferase assay as described above. The frequencies of genes in three groups were: E>200, 37.5%; 200>E>100, 12.5%; 100>E>30, 50%. The frequencies of siRNAs CEN in each group were: E>200, 83.3%; 200>E>100, 100%; 100>E>30, 37.5%. (**B**) Mutations were introduced into the target site in *IL-2* core promoter and generated the mutated *IL-2* promoter, IL-2mt-27/−25 or IL-2mt-17/−16. HEK293T cells were transfected with the wild-type or mutated *IL-2* promoter-driven reporter constructs and siRNA IL2-CEN or NC. Thirty-six hrs later, promoter activities were determined by dual-luciferase assay. (**C**–**F**) SiRNAs targeting the promoter of *IL-2*, *INS*, *CIRBP*, *APOE*, *BCL2L12*, *c-Myc*, *POMC* or *GAPD* were co-transfected into HEK293T cells with (**C**) *IL-2*, (**D**) *BCL2L12*, (**E**) *POMC* or (**F**) *c-Myc* promoter-driven reporter constructs. Thirty-six hrs later, promoter activities were determined by dual-luciferase assay. n = 3. **, *p*<0.01. ***, *p*<0.001.

In order to analyze the features of these activating siRNAs, we divided them into four groups according to the individual fold activation of the target gene promoter (E>200, 200>E>100, 100>E>30 or E<30, here E (%) stands for the enhancement of siRNA on promoter activity in percent compared with NC) (Table S1 in File S1). Among the most effective siRNAs in each group, 83.3% siRNAs in E>200 and 100% siRNAs in 200>E>100 were siRNAs CEN (Fig. 2A). It suggested that siRNAs CEN could activate gene promoters more effectively than siRNAs UP or DN.

The most effective siRNA for each gene was picked up and the promoter activities relative to NC were listed in Fig. 2A. We found that the promoter activities of all the tested genes could be upregulated by more than 30% with one TATA-box-targeting siRNA alone. Among them, the promoter activities of half of the genes could be enhanced by more than 100%, and the promoter activities of 37.5% genes could be enhanced by more than 200% (Fig. 2A). These data indicate that TATA-box-targeting siRNAs could become an efficient tool to sequence-specifically upregulate transcription of a target gene.

To investigate whether the promoter activation by the activating siRNAs is specific for the target gene, mutations were introduced into the target site in *IL-2* core promoter (Table 1). The luciferase reporters driven by either the wild-type *IL-2* promoter (IL-2-pro) or the mutated *IL-2* promoter (IL-2mt-27/−25 or IL-2mt-17/−16) were co-transfected with siRNA IL2-CEN into HEK293T cells. The dual-luciferase assay indicated that the siRNA IL2-CEN had no enhancement effect on the mutated promoters any longer, especially the mutation which impaired the binding in seed sequence (IL-2mt-17/−16) (Fig. 2B). Furthermore, we examined the effects of siRNAs targeting some gene promoters on other gene promoter activities. The result confirmed the specificity of the siRNA-promoter interaction (Fig. 2C–F).

The key RNAi components such as AGO proteins have been reported to participate in transcriptional regulation mediated by small non-coding RNAs in the nucleus [15], [54], and we have recently found that both AGO1 and AGO2 may be recruited to the *IL-2* promoter by let-7i for its regulation function (see the related manuscript). Therefore, it is intriguing to examine whether the regulation of the activating siRNAs is also through an AGO-dependent way. When AGO1 or AGO2 was knocked down by siRNAs, the activation of *IL-2* promoter transcription by the activating siRNA was significantly impaired (Fig. S1 in File S1), indicating that both AGO1 and AGO2 may be involved in the activation of gene promoter mediated by the activating siRNA.

**Figure 3:**
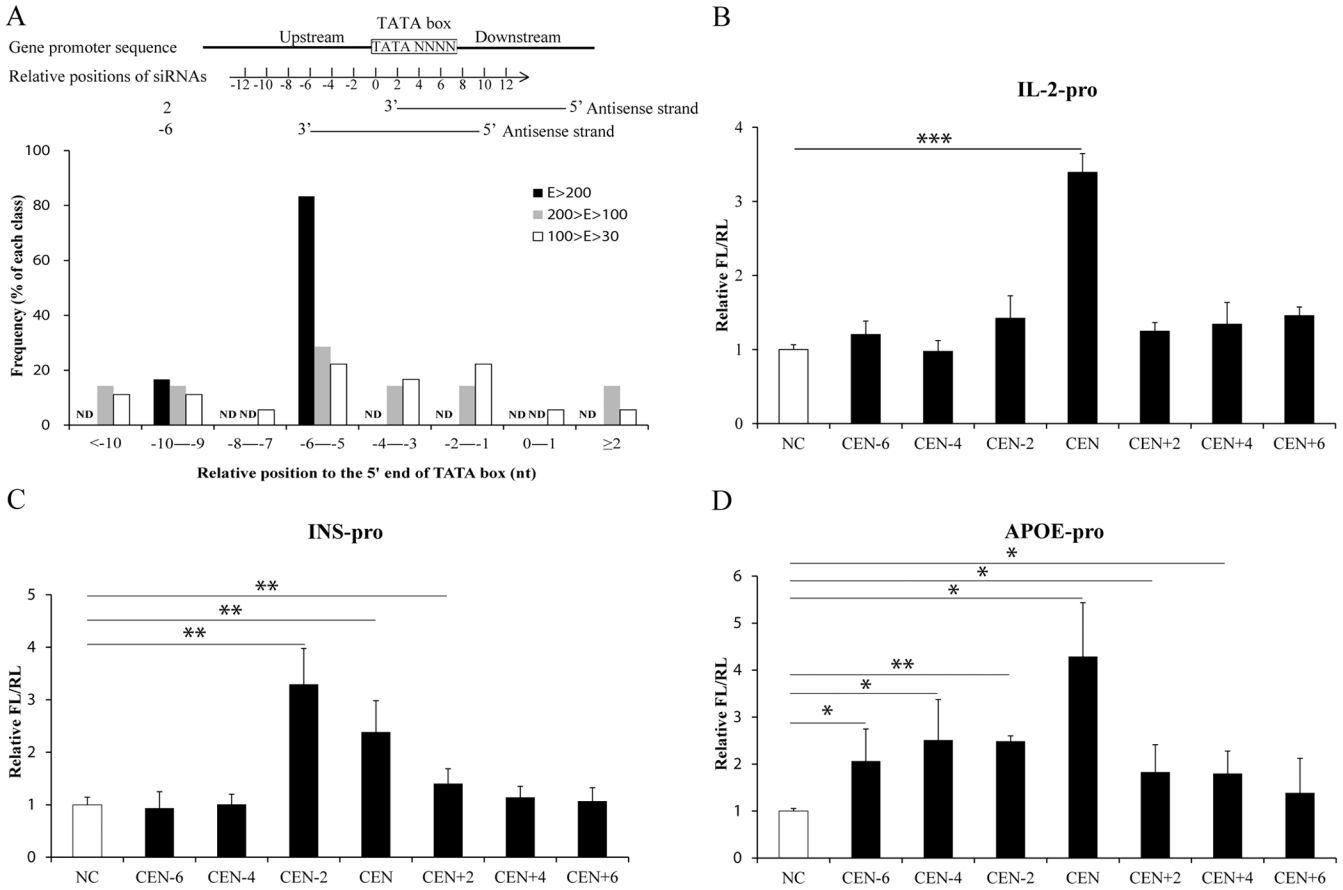
SiRNAs targeting the center of TATA-box motif upregulate gene promoter activities with high efficiency. (**A**) The frequency distributions of functional activating siRNAs targeting different positions relative to the TATA-box. The consensus sequence of TATA-box motif was highlighted in the schematic diagram, and relative positions of siRNAs were indicated by the distances between the 3′ ends of siRNAs antisense strands and the 5′ ends of TATA-box (position 0). For example, the relative position of siRNA whose 3′ end targeting the third nucleotide of the TATA-box was position 2, and the relative position of siRNA CEN whose 3′ end targeting the sixth or fifth nucleotide upstream the TATA-box was position −6 or −5 (top panel). According to the promoter activities determined by dual-luciferase assay, functional activating siRNAs were divided into three groups (E>200, black square; 200>E>100, gray square; and 100>E>30, white square. Here E (%) stands for the enhancement of siRNA on promoter activity in percent compared with NC). The frequency distributions of the siRNAs with different relative positions in each group were indicated. ND, not detected. (**B**–**D**) Promoter activity assay of (**B**) *IL-2*, (**C**) *INS* or (**D**) *APOE* promoter with siRNAs against different sites of the TATA-box motif. SiRNAs targeting various positions centered with the TATA-box motif were transfected and promoters activities were determined as described above. *P*-values were calculated using the two tailed unpaired Student’s t-test with equal variances. n = 3. *, *p*<0.05. **, *p*<0.01. ***, *p*<0.001.

Then the relative positions of the siRNAs to the TATA-box were measured by the distances between the 3′ ends of siRNA antisense strands and the 5′ ends of the TATA-box (Fig. 3A, top panel). We found that most functional activating siRNAs (siRNAs in E>200, 200>E>100 or 100>E>30) targeted the TATA-box-centered position (−6–−5 bp upstream the 5′ end of TATA-box motif) when compared with non-functional siRNAs (siRNAs in E<30) (Fig. 3A bottom, Fisher’s exact test, *p* = 0.019). To confirm this result, we chemically synthesized various siRNAs against different sites of human *IL-2*, *INS* or *APOE* promoter (Table 1) and performed transfection as described above. Similarly, dual-luciferase assay showed that siRNAs CEN upregulated the promoter activities of all three genes more powerfully than siRNAs targeting the positions shifted from the TATA-box-centered position (Fig. 3B–D). These data suggested that siRNAs targeted the TATA-box-centered position activate gene promoters with high efficiency.

### The sequence or length modifications of siRNAs are correlated to activation efficiency

**Figure 4:**
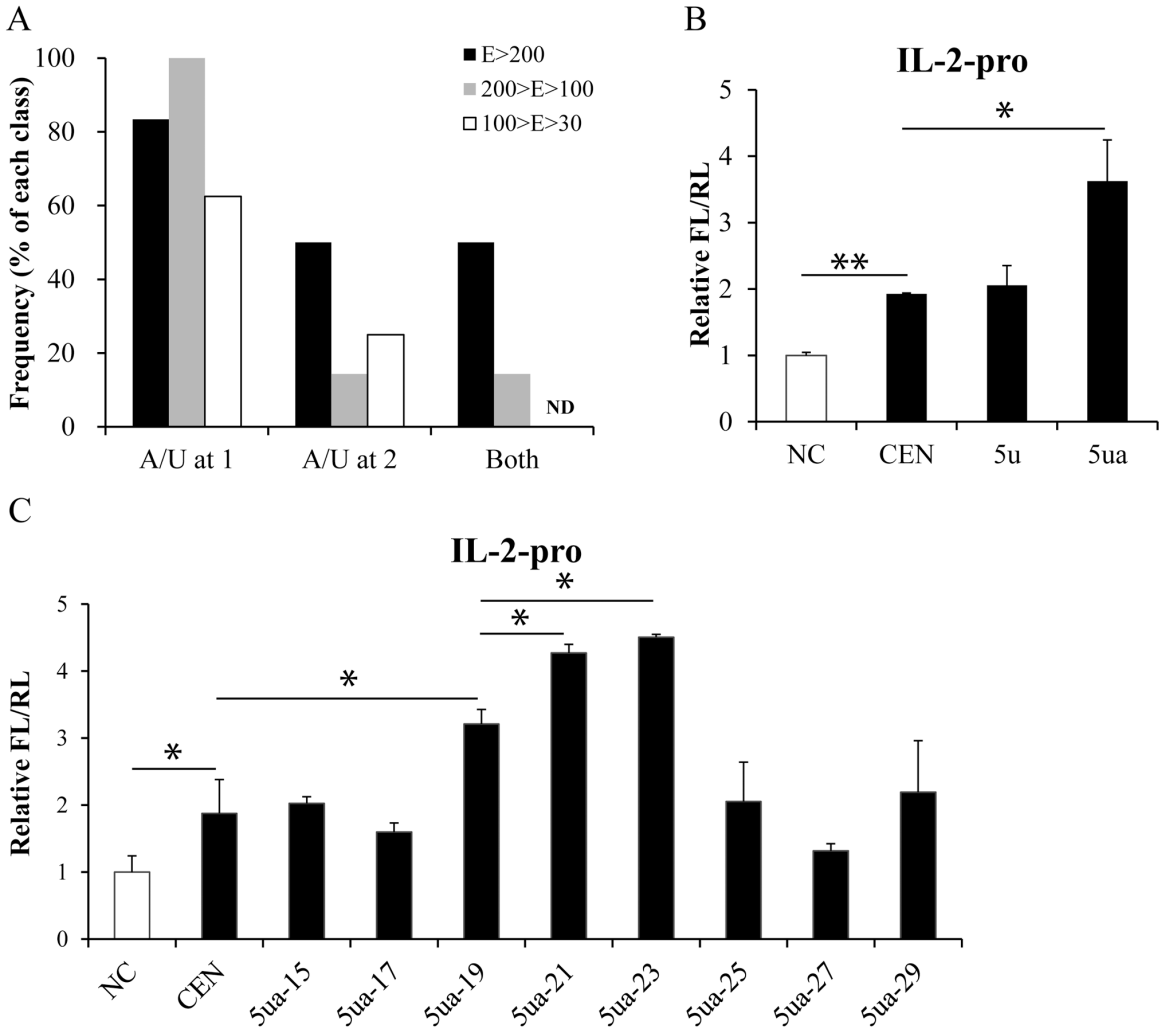
Sequence characters and length are correlated with the efficiency of siRNA-induced activation. (**A**) The frequency distributions of functional activating siRNAs with 1-A/U, 2-A/U or both in the antisense strand. ND, not detected. (**B**) Effects of sequence modification in the 5′ end of antisense strand on the siRNA-induced activation of *IL-2* promoter. 5u, the sequence-modified siRNA whose first base in 5′ end of antisense strand was substituted into U; 5ua, the sequence-modified siRNA whose first two bases in 5′ end of antisense strand were substituted into UA. (**C**) Effects of siRNAs with different lengths on *IL-2* promoter activity. 5ua-15, the length-modified siRNA 5ua whose length was 15 nts; 5ua-19, the length-modified siRNA 5ua whose length was traditional 19 nts; and so on. The promoter activities were determined with dual-luciferase assay as described above. *P*-values were calculated using the two tailed unpaired Student’s t-test with equal variances. n = 3. *, *p*<0.05. **, *p*<0.01. ***, *p*<0.001.

Several criteria have been suggested for designing efficient repressive siRNAs, such as low G/C content (30%–50%) and 1–3-A/U in the antisense strand [17]–[20]. To examine whether these features are also important for the function of activating siRNAs, their sequence characteristics were analyzed (Table S1 in File S1). The antisense strands of more than 60% activating siRNAs in E>200 or 100>E>30 had 30%–50% G/C content, but only 25% activating siRNAs in 200>E>100 showed 30%–50% G/C content (Fig. S2 in File S1). It seemed that the proportion of siRNAs with 30%–50% G/C content was not correlated to the activation efficiency in our study. Similarly, most siRNAs in all the three functional activating classes contained the 1-A/U nucleotide in the antisense strands, but the presence of A/U at the first two bases in the antisense strand was significantly more frequent in E>200 and 200>E>100 than in 100>E>30 (Fig. 4A). The low internal stability of the 5′ end would be helpful for siRNA duplex unwinding and the loading of antisense strand into RISC [27]. Subsequently, we tried to modify the siRNAs to enhance their abilities according to this feature. The first one or two bases were substituted into U (5u) or UA (5ua) in the antisense strand of siRNA CEN targeting the *IL-2* promoter. The normal and modified siRNAs were respectively transfected into HEK293T cells with reporter constructs and promoters activities were detected as described above. As expected, the sequence modified siRNAs 5ua displayed enhanced effects (Fig. 4B).

Although the well-accepted length of siRNAs is 19 nts, it remains uncertain whether the length of the activating siRNA is correlated to its efficiency. Several siRNAs with different lengths were synthesized based on the sequence modified as above (Table 1). Unexpectedly, the siRNA with 21 nts or 23 nts (5ua-21 or 5ua-23) activated *IL-2* promoter activity more potently than siRNAs with other lengths (Fig. 4C). Alternatively, previous studies suggested that ss-siRNAs with or without chemical modification could efficiently induce mRNA degradation [48]–[51]. To determine whether ss-siRNAs are efficient for activating transcription, the single-stranded sense or antisense strands of siRNAs that targeting *IL-2* promoter were synthesized and used to test their activating efficiency. The sequences of these single-stranded siRNAs were listed in Table S2 in File S1. However, neither of them showed any activating effect when comparing with the double-strand siRNAs (Fig. S3 in File S1). Taken together, these data demonstrated that sequence or length modified siRNAs robustly activated gene promoter activities.

### Sequence modifications of siRNAs for nuclear importing displayed no enhancement effect on gene promoter activities

Some groups found that miRNAs with the sequence ASUS (S = C/G) at their 3′ termini could accumulate in the nucleus [28]–[30]. In our study, TATA-box-targeting siRNAs are expected to function in the nucleus. Therefore the sequence ASUS or the relaxed motifs SUS, ASU or ASNS (N = A/G/C/U) in the 3′ regions of the antisense strands of the effective siRNAs were searched (Table S1 in File S1). Among the three functional activating classes, many siRNAs contained the relaxed motifs, but only 33% of the siRNAs in E>200 contained ASUS at their 3′ ends of the antisense strands (Fig. S4A in File S1). We hypothesized that introduction of this motif into siRNAs may improve their effects on gene promoter activities. SiRNAs targeting the TATA-box of each of three genes with modified nuclear import sequence (Mip) were synthesized (Table S3 in File S1). After siRNAs co-transfected with reporter constructs as described above or siRNAs transfected alone, promoters activities were detected and subcellular distribution of siRNAs were determined. Nevertheless, we didn’t find any significant improvement of neither the enrichment in the nucleus nor the activation of gene promoters (Fig. S4B–D in File S1). These may be respectively due to the different nuclear import mechanism determined by the sequence difference between siRNA and miRNA, or low demand for siRNAs to activate gene promoters within the nucleus.

### Both the 5′ end and 3′ end of antisense strand were important for the function of the activating siRNA

**Figure 5:**
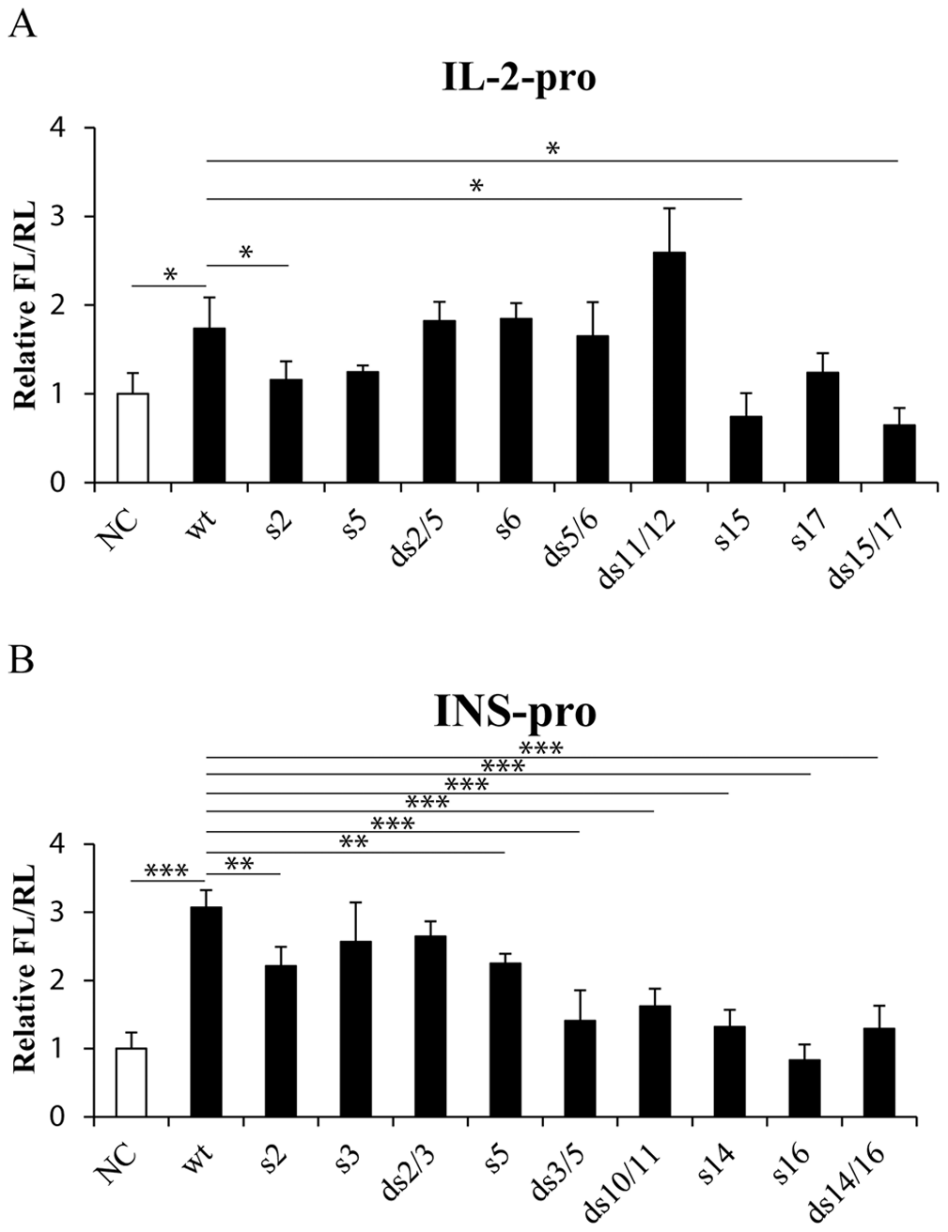
Mismatch tolerance of functional activating siRNAs. Promoter activities of (**A**) *IL-2* or (**B**) *INS* were determined with siRNAs harboring a serial mutations listed in Table 2. The promoter activities were determined with dual-luciferase assay as described above. *P*-values were calculated using the two tailed unpaired Student’s t-test with equal variances. n = 3. *, *p*<0.05. **, *p*<0.01. ***, *p*<0.001.

Amarzguioui *et al*. reported that the 3′ end of the antisense strand of siRNA hTF167i had a general tolerance to mutations (G/C transversions), while the 5′ end (within the seed region) exhibited low tolerance to mutations [55]. To explore how the mutations affect the efficiency of activating siRNAs, we designed nine single- or double-mutant siRNAs based on siRNAs CEN targeting *IL-2* or *INS* promoter and named them according to the mutated position relative to the 5′ end of the antisense strand (Table 2). According to the promoter activity alterations after transfection of the siRNAs mutants, the seed region of the antisense strands of siRNAs targeting *IL-2* or *INS* promoter showed low tolerance to mutations, whereas mutations in the 3′ ends impaired the function of the activating siRNAs more significantly (Fig. 5A, B). Meanwhile, when two bases in the TATA-box-binding region were mutated, the activation efficiency on *IL-2* promoter was not significantly impaired, but the activation efficiency on *INS* promoter was weakened (Fig. 5A, B). These data indicate that besides the traditional seed region, the binding of 3′ termini of the siRNAs to the target sequences is important for their activation effects.

### SiRNAs with 2′-OMe modification at the 3′ termini exhibit long-lasting efficacy

**Figure 6:**
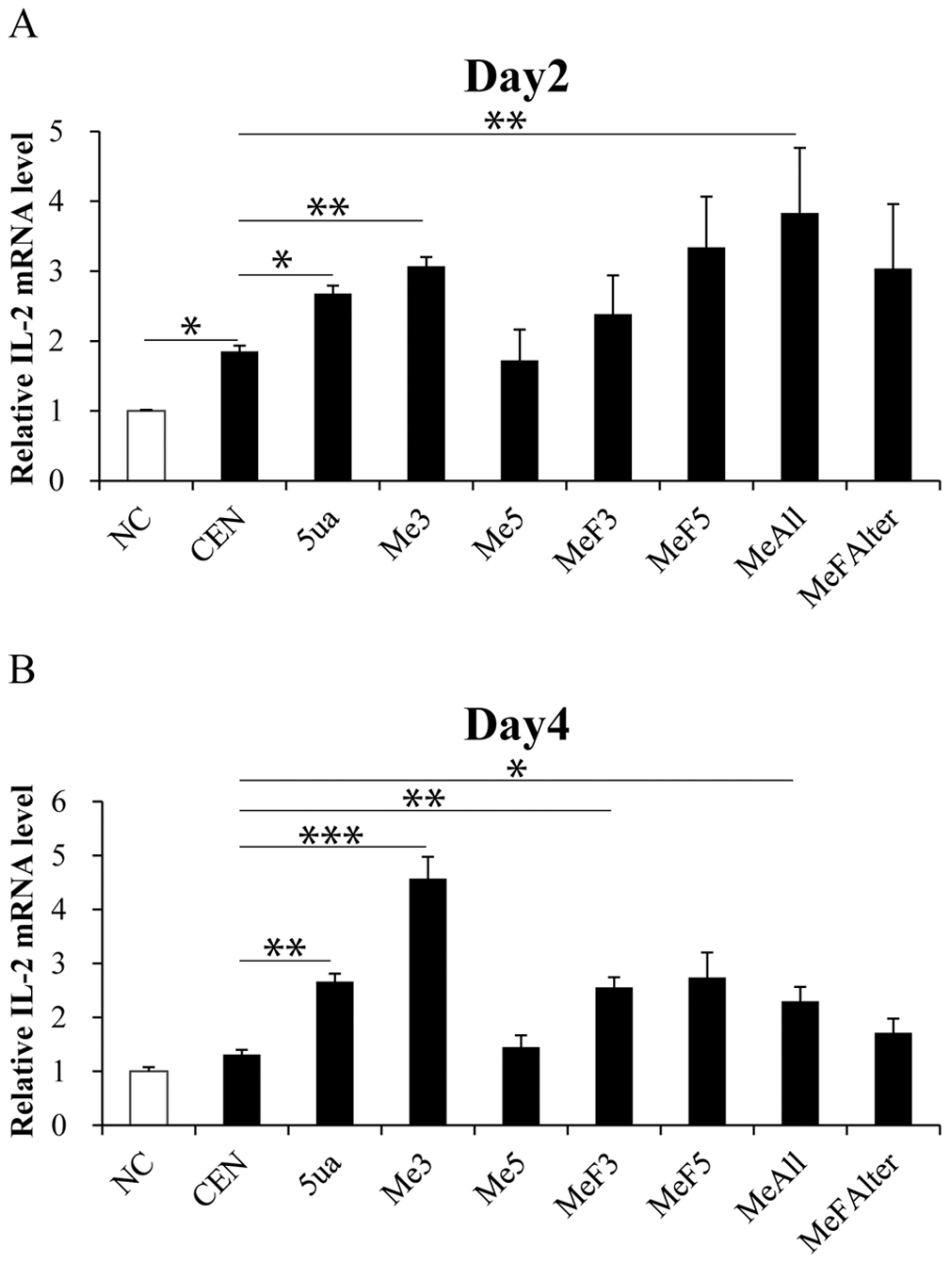
Certain chemical modifications enhance the siRNAs efficacy on gene promoter activation. **(A**–**B**) Effects of chemical modified siRNAs on IL-2 mRNA level in Jurkat cell line. 2–3×10^4^ Jurkat cells were transfected with 120 pmol siRNAs for 12 hrs, and were subsequently treated with PMA (50 ng/ml) and ionomycin (1 µM) till (**A**) 2 days or (**B**) 4 days post transfection. IL-2 mRNA levels were then evaluated by qRT-PCR and normalized to β-actin. *P*-values were calculated using the two tailed unpaired Student’s t-test with equal variances. n = 3. *, *p*<0.05.

To further investigate the effects of chemical modifications on the efficiency of siRNAs, 2′-OMe and 2′-F modifications were introduced to different nucleotides of siRNAs (Table 3). Jurkat cells were transfected with these modified siRNAs for 12 hrs, and were subsequently treated with *phorbolmyristate acetate* (PMA, 50 ng/ml) and ionomycin (1 µM). All the chemical modified siRNAs enhanced IL-2 mRNA expression in Jurkat cells at two days post transfection (Fig. 6A). However, when proceeded to four days, only the siRNA with four 2′-OMe modified bases in the 3′ termini (Me3) robustly upregulated IL-2 mRNA level (Fig. 6B). Given that chemical modification can reduce the off-target effects of siRNAs [41], [43], we examined whether other interleukins expressed in CD4^+^ T cells and Jurkat cells were affected. As expected, neither IL-4 nor IL-5 mRNA level was influenced by the modified or normal siRNAs targeting the *IL-2* TATA-box motif (Fig. S5A–D in File S1). These data suggest that chemical modifications can improve the stability of siRNAs and maintain the activation efficiency for longer time. Furthermore, the chemically-modified siRNAs also possessed good specificities.

### Optimized siRNAs exhibit improved potency and long-time effect on enhancing IL-2 expression in human and mouse primary CD4^+^ T cells

**Figure 7:**
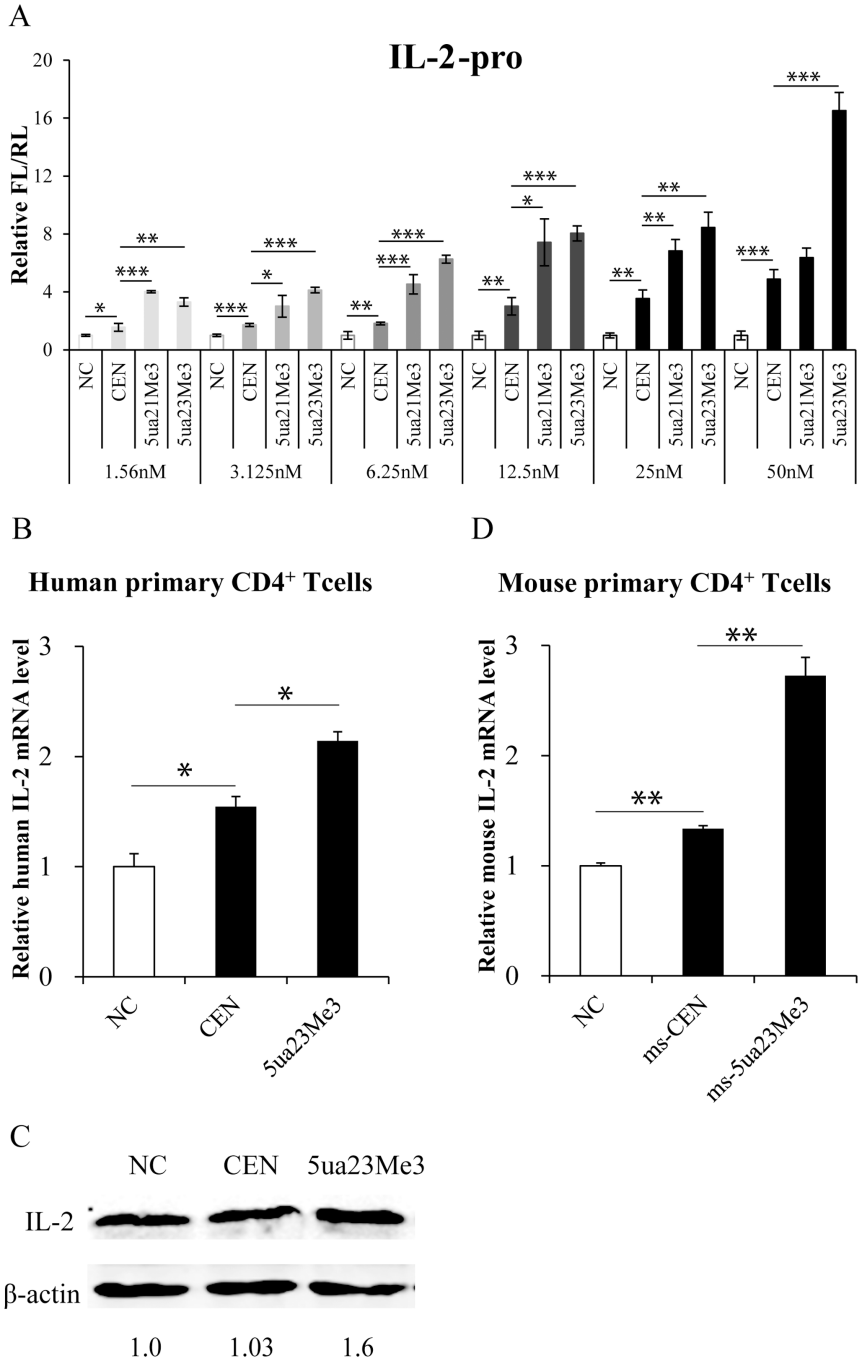
Optimized siRNAs showed higher and longer efficiency on *IL-2* promoter activation in human or mouse primary CD4^+^ T cells. **(A)** HEK293T cells were transfected with siRNAs at the indicated concentrations and promoters activities were determined as described above. **(B)** Effects of optimized siRNAs on IL-2 mRNA level in human primary CD4^+^ T cells. Human primary CD4^+^ T cells were transfected with 120 pmol siRNAs for 12 hrs, and were subsequently stimulated with anti-CD3 (1 µg/ml) and anti-CD28 (5 µg/ml) antibodies for 84 hrs. IL-2 mRNA levels were determined by qRT-PCR as described above. **(C)** Western blot analysis of IL-2 protein in human primary CD4^+^ T cells in (B). The β-actin was selected as an internal control. **(D)** Effects of optimized siRNAs on IL-2 mRNA level in mouse primary CD4^+^ T cells. Mouse primary CD4^+^ T cells were transfected with 120 pmol siRNAs for 12 hrs, and were subsequently stimulated with anti-mouse CD3 (2 µg/ml) and anti-mouse CD28 (1 µg/ml) antibodies for 84 hrs. Mouse IL-2 mRNA levels were determined by qRT-PCR and normalized to mouse GAPDH. *P*-values were calculated using the two tailed unpaired Student’s t-test with equal variances. *, *p*<0.05. **, *p*<0.01. ***, *p*<0.001. These data represented three independent experiments.

Taken together, we found four characteristics contributed to the high efficiency of TATA-box-targeting siRNAs on gene promoter activation: UA at the 3′ end of the antisense strand, 23 nts in length, targeting the center of TATA-box, and four 2′-OMe modified bases at the 3′ terminus of the antisense strand (Table 4). Then, we combined these characteristics and synthesized the optimized siRNAs targeting human or mouse *IL-2* promoter (Table 1, Table 3). The effects of optimized siRNAs on human *IL-2* promoters were compared with unmodified siRNAs in a dose-dependent experiment (Fig. 7A). Even at a very low concentration (1.56 nM), both optimized siRNAs, 5ua21Me3 and 5ua23Me3, upregulated *IL-2* promoter activities efficiently, while for the unmodified siRNA CEN, the concentration needed to be increased to 25 nM or 50 nM to achieve the similar efficacy. These optimized siRNAs showed about 16-fold improvement in potency over the unmodified siRNA. Moreover, at the concentration of 50 nM, 5ua23Me3 were more effective than 5ua21Me3, possibly because more complementary matches have stabilized the interaction between the siRNA and gene promoter.

**Table 4:** Contributions of five characteristics to the functionality of activating siRNAs.

Characteristics	Contribution to activating efficiency
TATA-box-centered target site	++
“UA” bases at the first two positions in antisense strand	++
23 nts in length	++
Mismatch in the 3′ region of the antisense strand	–
2′-OMe modification at the 3′ terminus of the antisense strand	+++

To examine the effects of the optimized siRNAs on the endogenous *IL-2* promoter activity in long time, we compared their effects with unmodified siRNAs in human or mouse primary CD4^+^ T cells. After transfection for 12 hrs and subsequent stimulation till 4 days, human or mouse primary CD4^+^ T cells were harvested for total RNAs extraction. Then human or mouse IL-2 mRNA levels were determined by quantitative real-time RT-PCR (qRT-PCR) and normalized to human β-actin and mouse GAPDH respectively. Human IL-2 protein levels were analyzed by Western blot and normalized to human β-actin. Compared to normal siRNAs, the optimized siRNA 5ua23Me3 enhanced IL-2 mRNA and protein expression potently in human CD4^+^ T cells (Fig. 7B, C), and the siRNA ms-5ua23Me3 enhanced IL-2 mRNA expression more potently in mouse CD4^+^ T cells (Fig. 7D). These prospective results could be due to the improved ability of being loaded to RISC and the enhanced stability of siRNAs with optimizations in sequence and length.

## Discussion

Generally, gene expression is regulated by the activation or deactivation of transcription factors after receiving signals from pathways triggered by ligand-receptor interactions [56]–[58]. Design of artificial transcription factors (ATFs) that mimic endogenous transcription factors (TFs) have been tried to modulate gene expression [59]–[64]. However, the regulation effects of TFs and ATFs are not so specific to affect a single specific gene. In this study, we showed that synthetic siRNAs targeting the TATA-box motif in gene promoters could activate these promoters effectively and specifically. Previous studies on agRNAs reported that the target sites of active or repressive siRNAs were located at about 1000 bp upstream [12] or overlapping (−9 to +2) the TSS [65]. In addition, siRNA siVFp (−992) inhibited the expression of human VEGF promoter even when the target site was deleted from the promoter, indicating that the inhibition was not occurring through specific targeting of the VEGF promoter [66]. The target sites from different studies were not always coincident. However, almost all the functional activating siRNAs in our study are targeting the TATA-box-centered position (∼34 bp upstream the TSS) (Fig. 3A).

TATA-box represents the most conserved and a wide-spread core promoter [67], [68]. TBP turnover on TATA-containing promoters is significantly higher than that on non-TATA promoters in yeast [69], indicating that it is a highly regulated process. Our previous study has shown that miRNA let-7i could directly interact with the TATA-box motif in *IL-2* promoter and is associated with TBP, implying the miRNA may affect the assembly of pre-initiation complexes (PICs) (see the related manuscript). The specific target sites of the functional activating siRNAs in our study may enable the TATA-box-targeting siRNAs to facilitate the recruitment of PICs members onto the TATA-box in gene promoters and subsequently promote the transcription.

In consistence with previous studies, the optimized siRNAs with U/A in the 5′ termini of antisense strands exerted enhanced activities (Fig. 4B), because the RISC complex prefer loading the siRNA with low energy end [27]. Although conventional siRNAs are 19 nts duplexes with two nucleotides at 3′ overhangs, our data demonstrated that 23 nts siRNAs with dTdT as the 3′ overhangs displayed most pronounced efficacy (Fig. 4C). The base pairing at 2–8th nucleotides of miRNA (seed region) is thought to be a key feature for the successful regulation of gene expression [70], [71]. Likewise, base pairs formed by the 5′ end of siRNA antisense strand are important for siRNA-target binding and cleavage [27], [35], [55], [72], [73]. After the cleavage of dsRNA to short 21–23 nts siRNA by Dicer, the RISC complex recognize and unwind the siRNA, then target and cleave the complementary mRNA by the binding of the 5′ end of antisense strand to target mRNA [22], [24], [74]. In addition to the importance of traditional “seed region” (Fig. 2B, Fig. 5), the results of our siRNA mutation experiment suggested that the binding of the 3′ region of the TATA-box-targeting siRNAs to target sequences was more essential for their activating function (Fig. 5).

The results above may be due to the different thermodynamic properties of the binding of RNA-RNA and RNA-DNA hybrids. RNA/RNA duplex is more stable than DNA/DNA double helix and RNA/DNA hybrid duplex with the same nearest-neighbor sequence [75]. Therefore, the effective binding of siRNAs to the TATA-box in gene promoter needs more complementary matches. This might explain the failing try of mutating the 3′ ends of invalid siRNAs into nuclear import sequence to strengthen their potency, which also weakened the binding between siRNAs and the target promoter sequences. Moreover, this observation may attribute to the different mechanisms of action among miRNAs, the traditional repressive siRNAs and the activating siRNAs. The differences are mainly due to three ways: first, the target of miRNAs and the traditional repressive siRNAs is RNA, while that of activating siRNA is DNA; second, the location for action of miRNAs and the traditional repressive siRNAs is in the cytoplasm, while activating siRNA regulate gene expression in the nucleus; third, the functional regulating components of miRNAs and the traditional repressive siRNAs are mainly AGOs and GW182 [76]–[79], while the associated proteins for activating siRNAs may also include general transcription factors such as Pol II, TBP, and TFIIB etc. (see the related manuscript), besides the AGO proteins (Fig. S1 in File S1).

It has been demonstrated that chemical modifications are well tolerated in the sense strands of suppressive siRNAs, whereas it is position-dependent tolerance for antisense strands modifications [80]. Therein, 2′-OMe/2′-F show positional effects on siRNAs activities, although they can be incorporated into both the sense and antisense strands [37], [41], [81]. For example, Prakash *et al*. showed that 2′-F sugar was generally well-tolerated on the antisense strand, whereas siRNAs with three 2′-OMe residues at the 3′ ends of the antisense strands were more active than the 5′-modified ones [37]. But Jackson *et al*. found that siRNAs with 2′-OMe modification at position 2 in the antisense strands can reduce off-target effects [41]. Our data showed similar results to that of Prakash *et al*., except that our functional activating siRNAs contained four 2′-OMe modified bases at the 3′ ends of the antisense strands (Fig. 6, Fig. 7). Presumably, these different results may be explained by conformational alterations of RNA-RNA and RNA-protein interactions mediated by chemical modifications at different position in siRNA sequences [41], [82], [83].

In conclusion, we have demonstrated that the siRNAs targeting the TATA-box-centered region effectively activated 14 out of 16 genes promoter activities, and the siRNA targeting TATA-box-centered region, with UA at the first two bases of the antisense strand, 23 nts in length, and with 2′-OMe modification at the 3′ terminus of the antisense strand, is most effective in activating gene transcription. Taken together with other reports, these results imply the potential of using appropriate modified siRNAs for technical or therapeutic applications. For instance, it is well known that some primary cells (for example, PBMCs) are very difficult to allow the entrance of transfected plasmids [84], [85]. When some genes need to be overexpressed in some situations, the specific siRNAs could be transfected into the primary cells to enhance the expression of target genes, which would be a simple and efficient experimental tool. Moreover, the application of TATA-box-targeting siRNAs to specifically activate some important genes, such as insulin, tumor repressor genes (*p21*) or DNA repair genes (*BRCA2*), may provide a new safe treatment strategy for many diseases such as cancer and diabetes. Further studies should be conducted to find the target position(s) and the sequence characteristics of activating siRNAs that could enhance the promoter activities of the genes without the TATA-box motif.

## Supporting Information

File S1 **Supporting Figures and Tables. Table S1. Feature analysis of siRNAs.** For each siRNA, only antisense strand is shown. Efficiency was the FL/RL (FL, firefly luciferase; RL, renilla luciferase) ratio relative to the negative control siRNA (NC) for each siRNA. SiRNAs were classified according to their individual fold upregulation of the efficiency in percent (E (%)) compared with NC. Relative position was the distance of 3′ end of siRNA antisense strand to 5′ end of TATA box. Nuclear import sequences in the 3′ region of antisense strand were in bold. S-G/C, N-A/G/C/U. **Table S2. Sequences of single-stranded oligonucleotides targeting human** ***IL-2*** **promoter and negative control.** Nucleotides in bold were corresponding to the TATA-box motif. Modified bases were in bold lowercase. ss, single strand; as, antisense; s, sense. **Table S3. Sequences of siRNAs targeting the TATA-box of** ***c-Myc*** **,** ***NPPA*** **, or** ***IL-6*** **with or without modified nuclear import sequence (Mip).** Modified bases were in lowercase. Nuclear import sequences in the 3′ region of the antisense strand of modified siRNA were in bold. **Figure S1. Effects of inhibiting AGO1, AGO2 or both genes on the activation of** ***IL-2*** **promoter by siRNA.** After transfection of 50 nM siRNAs for AGO1or AGO2 or the negative control siRNA (NC) into HEK293T cells for 24 h (1st transfection), 25 nM siRNA IL-2-CEN or NC were co-transfected with *IL-2* promoter-driven firefly luciferase (FL) and renilla luciferase (RL) constructs (2nd transfection). Thirty-six hrs later, promoter activities were determined by dual-luciferase assay. *P*-values were calculated using the two tailed unpaired Student’s t-test with equal variances. n = 3. *, *p*<0.05. **, *p*<0.01. ***, *p*<0.001. **Figure S2. Distribution of functional activating siRNAs with low G/C content.** The frequency of siRNAs with low G/C content (30%<G/C<50%) in the antisense strand in each functional activating class (E>200, 200>E>100, 100>E>30). **Figure S3.** **Impacts of ssRNAs (single strand RNAs) on** ***IL-2*** **promoter activity.** SiRNAs were co-transfected into HEK293T cells with the target promoter-driven firefly luciferase (FL) and renilla luciferase (RL) constructs. Thirty-six hrs later, promoter activities were determined by dual-luciferase assay. ss-asRNA, single strand antisense RNA; ss-sRNA, single strand sense RNA. *P*-values were calculated using the two tailed unpaired Student’s t-test with equal variances. n = 3. *, *p*<0.05. **, *p*<0.01. ***, *p*<0.001. **Figure S4. Nuclear import sequence modified siRNAs showed no significant improvement of neither the enrichment in the nucleus nor the activation of gene promoters.** (**A**) Analysis of reported nuclear import sequence in 3′ ends of siRNAs antisense strands. S-G/C, N-A/G/C/U. ND, not detected. Subcellular distributions of antisense strands of siRNAs with (middle) or without (left) modified nuclear import sequence (Mip) targeting (**B**) *c-Myc*, (**C**) *NPPA* or (**D**) *IL-6* promoter. Thirty-six hrs after siRNAs were transfected, nuclear and cytoplasmic fractions were separated. The expression levels of siRNAs, tRNA-Lys and U78 were then tested with qRT-PCR. Effects of siRNAs on gene promoter activities were determined with dual-luciferase assay as described above (right). *P*-values were calculated using the two tailed unpaired Student’s t-test with equal variances. n = 3. *, *p*<0.05. **, *p*<0.01. **Figure S5. The mRNA levels of IL-4 and IL-5 were not affected.** “On target” and “Off target” activities. At 12 hrs post transfection of 120 pmol siRNAs into Jurkat cells, cells were subsequently treated with PMA (50 ng/ml) and ionomycin (1 µM) for (**A**–**B**) 2 days or (**C**–**D**) 4 days. The mRNA levels of IL-4 and IL-5 were determined by qRT-PCR as described above. *P*-values were calculated using the two tailed unpaired Student’s t-test with equal variances. n = 3. *, *p*<0.05.
(DOCX)
