# Supplementary material for: Optimizations of SiRNA Design for the Activation of Gene Transcription by Targeting the TATA-Box Motif
Source: PLoS One. 2014 Sep 24;9(9):e108253. doi: 10.1371/journal.pone.0108253 (PMC4176967; doi:10.1371/journal.pone.0108253)
Supplement: File S1 — Supporting Figures and Tables. Table S1. Feature analysis of siRNAs. For each siRNA, only antisense strand is shown. Efficiency was the FL/RL (FL, firefly luciferase; RL, renilla luciferase) ratio relative to the negative control siRNA (NC) for each siRNA. SiRNAs were classified according to their individual fold upregulation of the efficiency in percent (E (%)) compared with NC. Relative position was the distance of 3′ end of siRNA antisense strand to 5′ end of TATA box. Nuclear import sequences in the 3′ region of antisense strand were in bold. S-G/C, N-A/G/C/U. Table S2. Sequences of single-stranded oligonucleotides targeting human IL-2 promoter and negative control. Nucleotides in bold were corresponding to the TATA-box motif. Modified bases were in bold lowercase. ss, single strand; as, antisense; s, sense. Table S3. Sequences of siRNAs targeting the TATA-box of c-Myc , NPPA , or IL-6 with or without modified nuclear import sequence (Mip). Modified bases were in lowercase. Nuclear import sequences in the 3′ region of the antisense strand of modified siRNA were in bold. Figure S1. Effects of inhibiting AGO1, AGO2 or both genes on the activation of IL-2 promoter by siRNA. After transfection of 50 nM siRNAs for AGO1or AGO2 or the negative control siRNA (NC) into HEK293T cells for 24 h (1st transfection), 25 nM siRNA IL-2-CEN or NC were co-transfected with IL-2 promoter-driven firefly luciferase (FL) and renilla luciferase (RL) constructs (2nd transfection). Thirty-six hrs later, promoter activities were determined by dual-luciferase assay. P-values were calculated using the two tailed unpaired Student’s t-test with equal variances. n = 3. *, p<0.05. **, p<0.01. ***, p<0.001. Figure S2. Distribution of functional activating siRNAs with low G/C content. The frequency of siRNAs with low G/C content (30%<G/C<50%) in the antisense strand in each functional activating class (E>200, 200>E>100, 100>E>30). Figure S3. Impacts of ssRNAs (single strand RNAs) on IL-2 promoter [file pone.0108253.s001.docx]

Supporting Information for

**Optimizations of SiRNA Design for the Activation of Gene Transcription by Targeting the TATA-box Motif**

Miaomiao Fan, Yijun Zhang, Zhuoqiong Huang, Jun Liu, Xuemin Guo, Hui Zhang*, and Haihua Luo*

File S1 includes:

Supporting Data (Table S1 to S3, Figure S1 to S5)

Supporting Methods

**Supporting Data**

**Table S1. Feature analysis of siRNAs**

| **Class** | **siRNAs** | **Antisense strand (5'-3')** | **Efficiency** | **Relative**  **position** | **G/C %** | **A/U at 1/2** | | | **Nuclear import sequence in the 3' region** | | | |
| --- | --- | --- | --- | --- | --- | --- | --- | --- | --- | --- | --- | --- |
|  |  |  |  |  |  | at 1 | at 2 | Both | ASUS | SUS | ASU | ASNS |
| **E>200** | IL-2-CEN | AGAUGCAAUUUAU**ACUG**UU | 3.392 | -5 | 26.32 | 5 | 3 | 3 | 2 | 4 | 3 | 3 |
|  | BCL2L12-CEN | UACAAACUUUAUU**AGU**UCG | 3.381 | -5 | 26.32 |  |  |  |  |  |  |  |
|  | INS-CEN | CGCUGGCUUUAU**AGUC**UCA | 3.278 | -6 | 47.37 |  |  |  |  |  |  |  |
|  | CIRBP-UP | UCUUAUAUACGCUUCCGCC | 3.182 | -10 | 47.37 |  |  |  |  |  |  |  |
|  | LHB-CEN | UAUCUGGCUAUAUAC**CUC**G | 3.161 | -5 | 42.11 |  |  |  |  |  |  |  |
|  | APOE-CEN | UUGUCCAAUUAU**AGGGCUC** | 3.009 | -6 | 42.11 |  |  |  |  |  |  |  |
| **200>E>100** | BCL2L12-DN | UCGUACAAACUUUAUUAGG | 2.810 | -2 | 31.58 | 7 | 1 | 1 | 0 | 1 | 2 | 1 |
|  | POMC-CEN | UCUGUCCUUAUAU**ACU**UGC | 2.657 | -5 | 36.84 |  |  |  |  |  |  |  |
|  | BCL2L12-UP | ACUUUAUUAGUUCA**ACU**UG | 2.544 | -10 | 26.32 |  |  |  |  |  |  |  |
|  | IL-2-UP | UAUACUGUUAAUU**CUG**GAA | 2.306 | -15 | 26.32 |  |  |  |  |  |  |  |
|  | IL-6-CEN | UGGAAACCUUAUUAAGAUU | 2.256 | -5 | 26.32 |  |  |  |  |  |  |  |
|  | CIRBP-DN | UCCCCGAGCCCGGCCUUAG | 2.175 | 3 | 73.68 |  |  |  |  |  |  |  |
|  | CIRBP-CEN | AGCCCGGCCUUAUAU**ACGC** | 2.167 | -3 | 57.89 |  |  |  |  |  |  |  |
| **100>E>30** | RHO-CEN | UCCCCAGACCCUUAUAAAG | 1.835 | -3 | 47.37 | 12 | 4 | 0 | 0 | 0 | 1 | 7 |
|  | IL-2-DN | GAACUCUUGAACA**AGAG**AU | 1.801 | 10 | 36.84 |  |  |  |  |  |  |  |
|  | APOE-DN | AGACUUGUCCAAUUAUAGG | 1.793 | -2 | 36.84 |  |  |  |  |  |  |  |
|  | NPPA-CEN | CGCCUCUUUUUAUAGCCCC | 1.791 | -5 | 52.63 |  |  |  |  |  |  |  |
|  | IL-6-DN | UGAUUGGAAACCUUAUUAG | 1.776 | -1 | 31.58 |  |  |  |  |  |  |  |
|  | c-Myc-DN | CAGACCCUCGCAUUAUAAA | 1.771 | -2 | 42.11 |  |  |  |  |  |  |  |
|  | H4k-UP | UCUUUAUACGAC**AGU**UGGC | 1.747 | -11 | 42.11 |  |  |  |  |  |  |  |
|  | HBB-DN | UCUGCCCUGACUUUUAUGC | 1.678 | -1 | 47.37 |  |  |  |  |  |  |  |
|  | INS-UP | CUUUAUAGUCUCAG**AGCC**C | 1.569 | -12 | 47.37 |  |  |  |  |  |  |  |
|  | HIV-CEN | CAGCUGCUUAUAUGUAGCA | 1.568 | -5 | 42.11 |  |  |  |  |  |  |  |
|  | GAPD-DN | UGCUCAAUUUAUAGAAACC | 1.523 | -6 | 31.58 |  |  |  |  |  |  |  |
|  | c-Myc-CEN | ACCCUCGCAUUAUAA**AGGG** | 1.512 | -5 | 47.37 |  |  |  |  |  |  |  |
|  | c-Myc-UP | UCGCAUUAUAA**AGGG**CCGG | 1.471 | -9 | 52.63 |  |  |  |  |  |  |  |
|  | CALCA-UP | UGCUCUUAUUCCCGCCGCU | 1.470 | -9 | 57.89 |  |  |  |  |  |  |  |
|  | INS-DN | GGCCCCCGCUGGCUUUAUA | 1.381 | 0 | 63.16 |  |  |  |  |  |  |  |
|  | HBB-CEN | UGCCCUGACUUUUAUGCCC | 1.340 | -3 | 52.63 |  |  |  |  |  |  |  |
|  | HBB-UP | UGACUUUUAUGCCC**AGCC**C | 1.321 | -8 | 52.63 |  |  |  |  |  |  |  |
|  | H4k-CEN | AGGCAGCGCCUUUAU**ACGG** | 1.313 | -3 | 57.89 |  |  |  |  |  |  |  |
| **E<30** | POMC-DN | UCCUCUGUCCUUAUAUACG | 1.295 | -2 | 42.11 | 13 | 2 | 2 | 0 | 4 | 0 | 2 |
|  | LHB-DN | UCGUGUAUCUGGCUAUAUG | 1.254 | 0 | 42.11 |  |  |  |  |  |  |  |
|  | APOE-UP | UCCAAUUAUAGGG**CUC**CCC | 1.193 | -9 | 52.63 |  |  |  |  |  |  |  |
|  | CALCA-DN | UCCAGCGACUG**CUC**UUAUG | 1.153 | 0 | 52.63 |  |  |  |  |  |  |  |
|  | H4k-DN | UGAGGCAGCGCCUUUAUAC | 1.121 | -1 | 52.63 |  |  |  |  |  |  |  |
|  | NPPA-DN | UGCCGCCUCUUUUUAUAGC | 1.100 | -2 | 47.37 |  |  |  |  |  |  |  |
|  | CALCA-CEN | AGCGACUGCUCUUAUUCCC | 1.090 | -3 | 52.63 |  |  |  |  |  |  |  |
|  | HIV-UP | UGCUUAUAUGUAGCAU**CUG** | 1.051 | -9 | 36.84 |  |  |  |  |  |  |  |
|  | GAPD-CEN | UGCGGGCUCAAUUUAUAGG | 0.896 | -2 | 47.37 |  |  |  |  |  |  |  |
|  | RHO-UP | ACCCUUAUAAA**GUG**AC**CUC** | 0.845 | -10 | 42.11 |  |  |  |  |  |  |  |
|  | HIV-DN | AAGCAGCUGCUUAUAUGUA | 0.843 | -2 | 36.84 |  |  |  |  |  |  |  |
|  | RHO-DN | UGACCCCCCCAG**ACCC**UUG | 0.841 | 2 | 68.42 |  |  |  |  |  |  |  |
|  | GAPD-UP | AAUUUAUAGAA**ACCG**GGGG | 0.569 | -11 | 42.11 |  |  |  |  |  |  |  |

For each siRNA, only antisense strand is shown. Efficiency was the FL/RL (FL, firefly luciferase; RL, renilla luciferase) ratio relative to the negative control siRNA (NC) for each siRNA. SiRNAs were classified according to their individual fold upregulation of the efficiency in percent (E (%)) compared with NC. Relative position was the distance of 3' end of siRNA antisense strand to 5' end of TATA box. Nuclear import sequences in the 3' region of antisense strand were in bold. S-G/C, N-A/G/C/U.

**Table S2. Sequences of single-stranded oligonucleotides targeting human *IL-2* promoter and negative control**

| **Human *IL-2* promoter sequence** |  |
| --- | --- |
| -50 to +1 | TAATATTTTTCCAGAATTAACAG**TATAAATT**GCATCTCTTGTTCAAGAGTTC |
| **ssRNA sense strand(5'-3')** |  |
| ss-NC | UUUGUACUACACAAAAGUACUG |
| IL-2-as-5ua | **ua**AUGC**AAUUUAUA**CUGUU-*dTdT* |
| IL-2-as-5ua21 | **ua**AUGC**AAUUUAUA**CUGUUAG-*dTdT* |
| IL-2-s-CEN | AACAG**UAUAAAUU**GCAUCU-*dTdT* |
| IL-2-s-5ua21 | CUAACAG**UAUAAAUU**GCAU**ua**-*dTdT* |

Nucleotides in bold were corresponding to the TATA-box motif. Modified bases were in bold lowercase. ss, single strand; as, antisense; s, sense.

**Table S3. Sequences of siRNAs targeting the TATA-box of *c-Myc*, *NPPA*, or *IL-6* with or without modified nuclear import sequence (Mip)**

| c-Myc-CEN | 5’-CCCUUUAUAAUGCGAGGGU-dTdT-3’ |
| --- | --- |
|  | 3’-dTdT-GGGAAAUAUUACGCUCCCA-5’ |
| c-Myc-CEN-Mip | 5’-gaCUUUAUAAUGCGAGGGU-dTdT-3’ |
|  | 3’-dTdT-**cuGA**AAUAUUACGCUCCCA-5’ |
| NPPA-CEN | 5’-GGGGCUAUAAAAAGAGGCG-dTdT-3’ |
|  | 3’-dTdT-CCCCGAUAUUUUUCUCCGC-5’ |
| NPPA-CEN-Mip | 5’-aacaCUAUAAAAAGAGGCG-dTdT-3’ |
|  | 3’-dTdT-uu**guGA**UAUUUUUCUCCGC-5’ |
| IL-6-CEN | 5’-AAUCUUAAUAAGGUUUCCA-dTdT-3’ |
|  | 3’-dTdT-UUAGAAUUAUUCCAAAGGU-5’ |
| IL-6-CEN-Mip | 5’-AAcacUAAUAAGGUUUCCA-dTdT-3’ |
|  | 3’-dTdT-UU**gugA**UUAUUCCAAAGGU-5’ |

Modified bases were in lowercase. Nuclear import sequences in the 3' region of the antisense strand of modified siRNA were in bold.


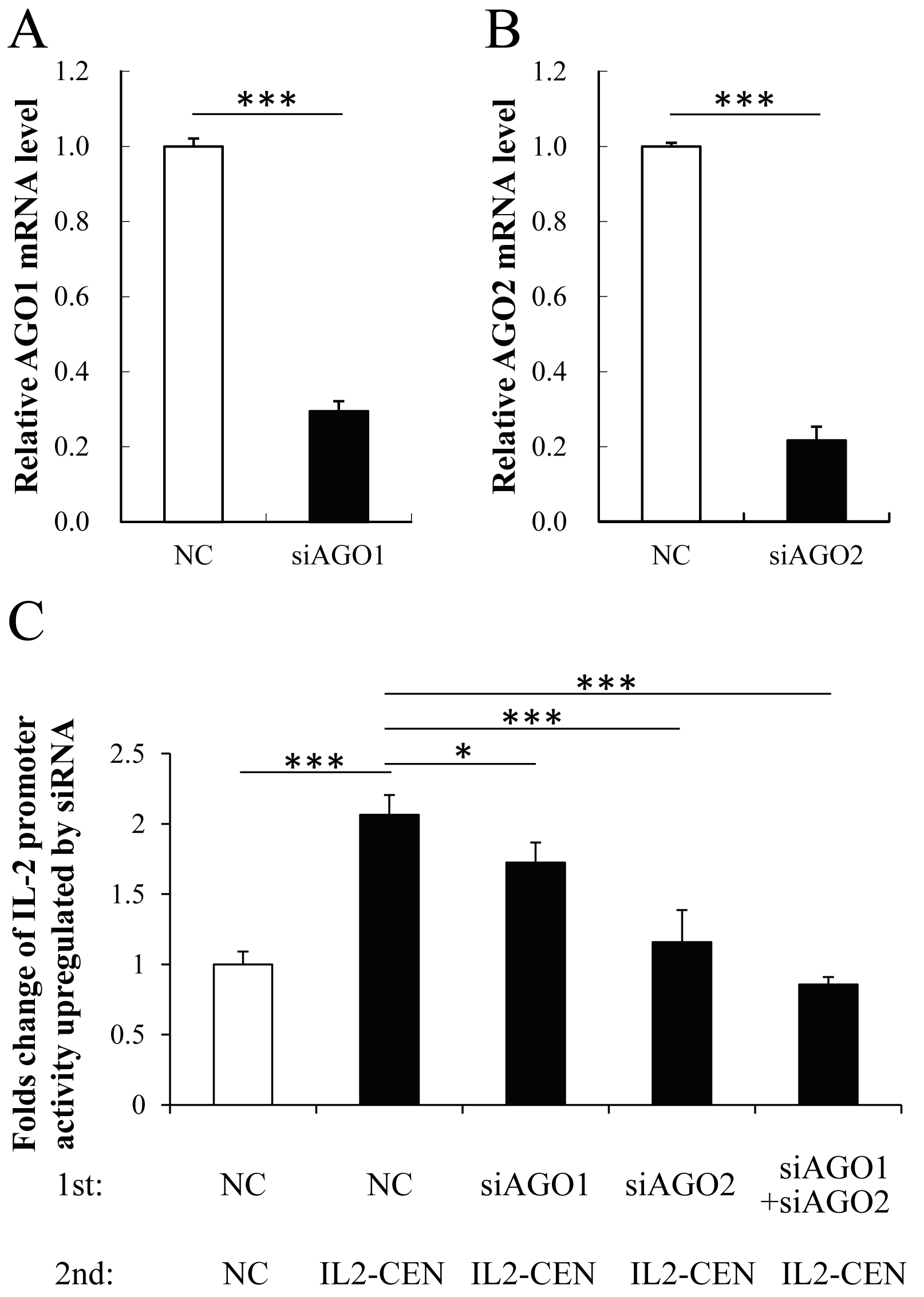


**Figure S1. Effects of inhibiting AGO1, AGO2 or both genes on the activation of *IL-2* promoter by siRNA.**

After transfection of 50 nM siRNAs for AGO1or AGO2 or the negative control siRNA (NC) into HEK293T cells for 24 h (1st transfection), 25 nM siRNA IL-2-CEN or NC were co-transfected with *IL-2* promoter-driven firefly luciferase (FL) and renilla luciferase (RL) constructs (2nd transfection). Thirty-six hrs later, promoter activities were determined by dual-luciferase assay. *P*-values were calculated using the two tailed unpaired Student’s t-test with equal variances. n=3. *, *p*<0.05. **, *p*<0.01. ***, *p*<0.001.

**
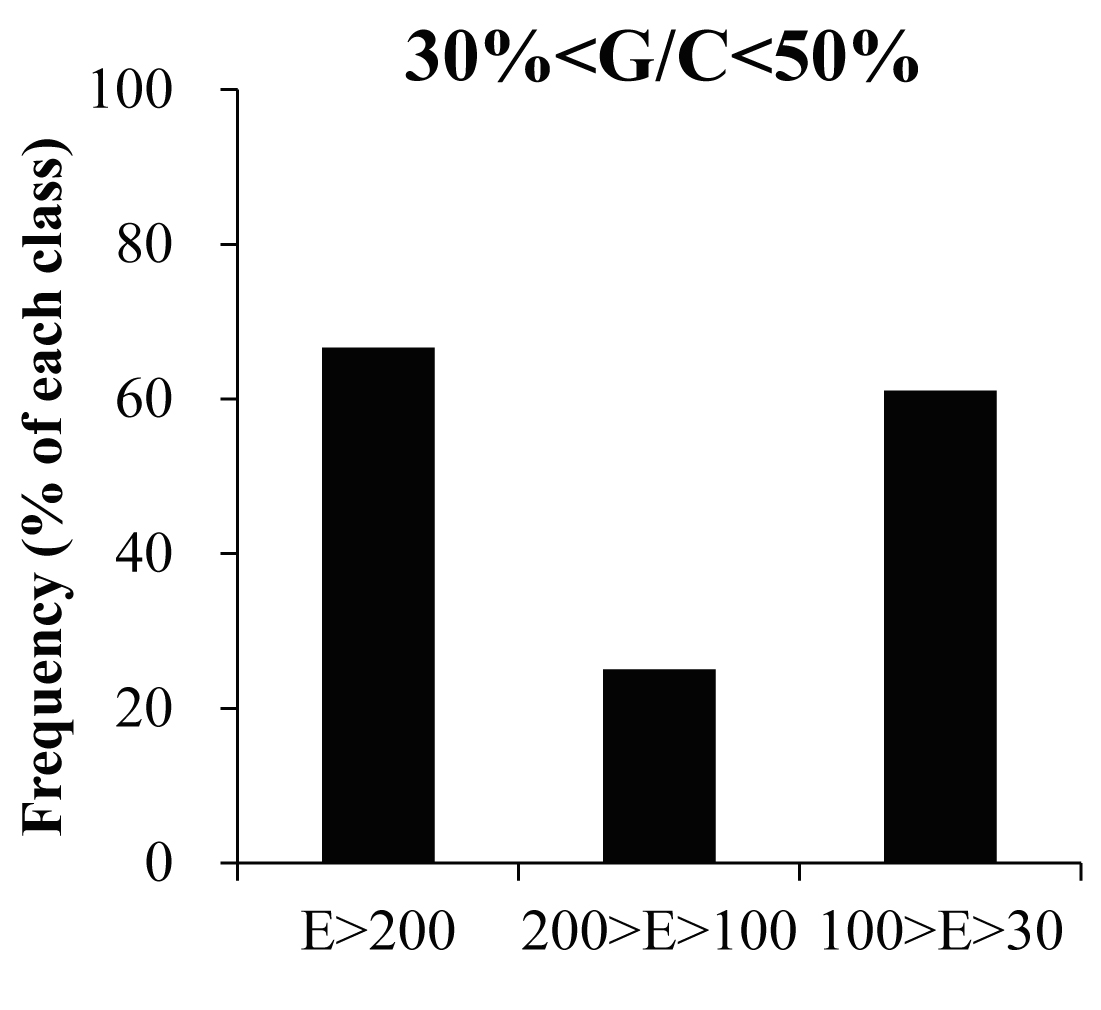
**

**Figure S2. Distribution of functional activating siRNAs with low G/C content.**

The frequency of siRNAs with low G/C content (30%<G/C<50%) in the antisense strand in each functional activating class (E>200, 200>E>100, 100>E>30).


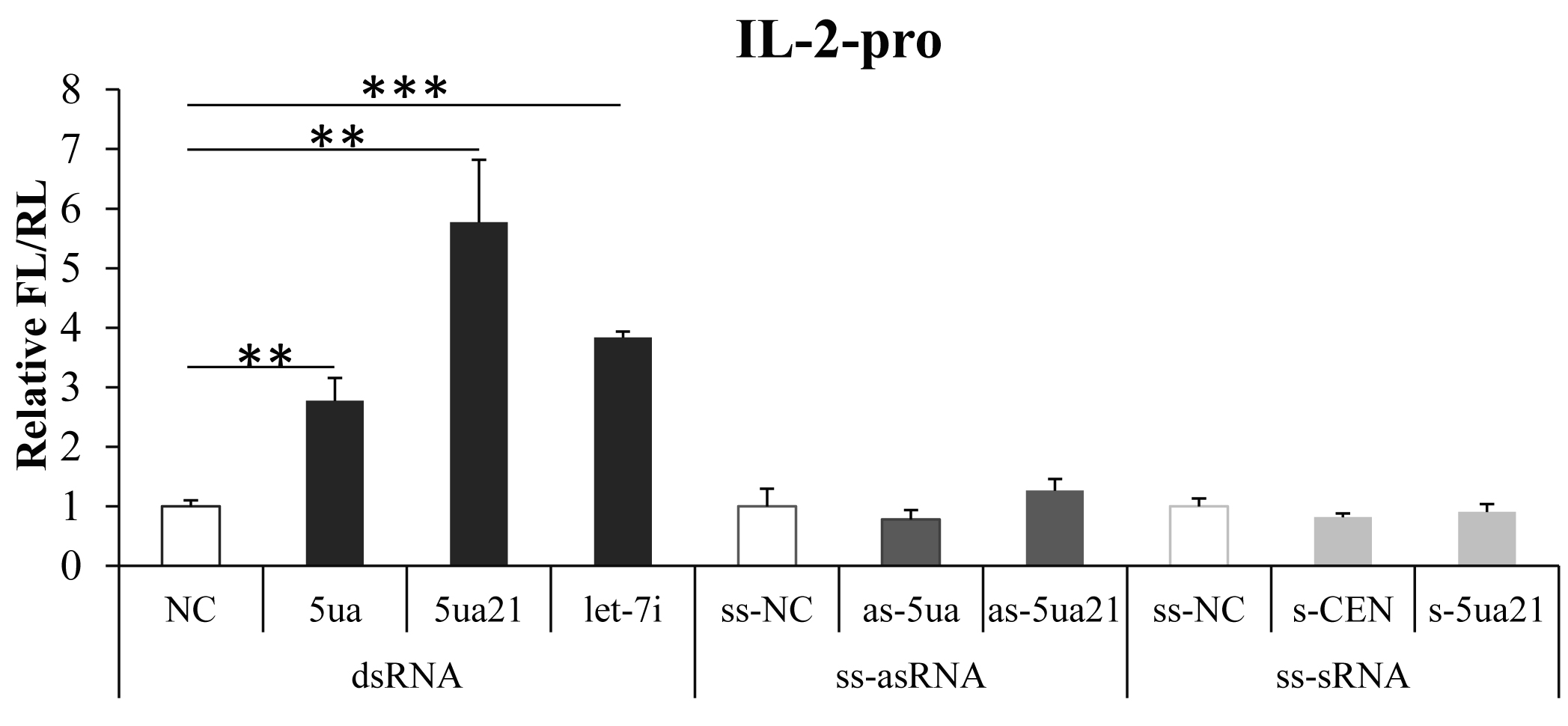


**Figure S3.** **Impacts of ssRNAs (single strand RNAs) on *IL-2* promoter activity.**

SiRNAs were co-transfected into HEK293T cells with the target promoter-driven firefly luciferase (FL) and renilla luciferase (RL) constructs. Thirty-six hrs later, promoter activities were determined by dual-luciferase assay. ss-asRNA, single strand antisense RNA; ss-sRNA, single strand sense RNA. *P*-values were calculated using the two tailed unpaired Student’s t-test with equal variances. n=3. *, *p*<0.05. **, *p*<0.01. ***, *p*<0.001.


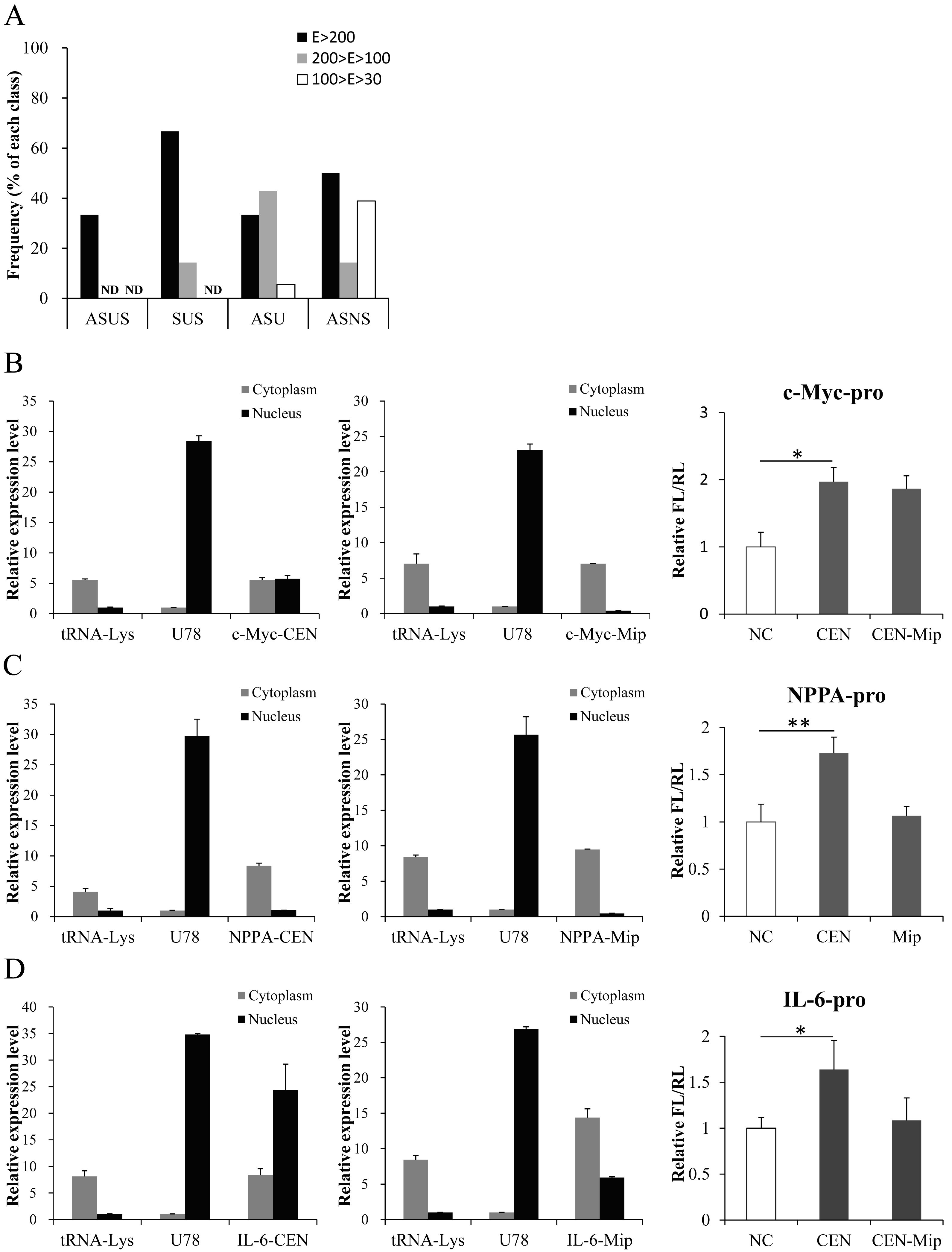


**Figure S4. Nuclear import sequence modified siRNAs showed no significant improvement of neither the enrichment in the nucleus nor the activation of gene promoters.**

(**A**) Analysis of reported nuclear import sequence in 3' ends of siRNAs antisense strands. S-G/C, N-A/G/C/U. ND, not detected. Subcellular distributions of antisense strands of siRNAs with (middle) or without (left) modified nuclear import sequence (Mip) targeting (**B**) *c-Myc*, (**C**) *NPPA* or (**D**) *IL-6* promoter. Thirty-six hrs after siRNAs were transfected, nuclear and cytoplasmic fractions were separated. The expression levels of siRNAs, tRNA-Lys and U78 were then tested with qRT-PCR. Effects of siRNAs on gene promoter activities were determined with dual-luciferase assay as described above (right). *P*-values were calculated using the two tailed unpaired Student’s t-test with equal variances. n=3. *, *p*<0.05. **, *p*<0.01.


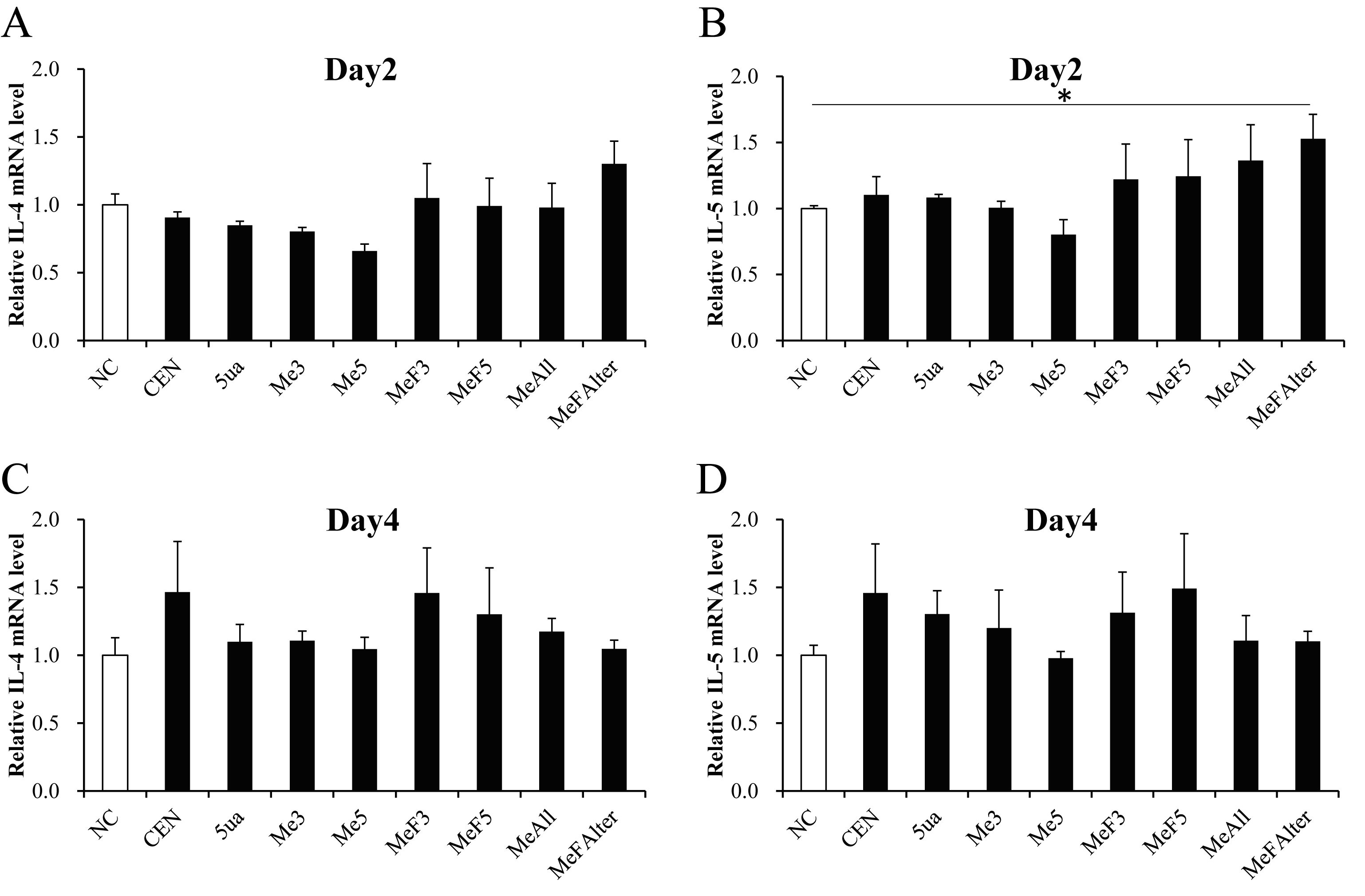


**Figure S5. The mRNA levels of IL-4 and IL-5 were not affected.**

"On target" and "Off target" activities. At 12 hrs post transfection of 120 pmol siRNAs into Jurkat cells, cells were subsequently treated with PMA (50 ng/ml) and ionomycin (1 μM) for (**A**–**B**) 2 days or (**C**–**D**) 4 days. The mRNA levels of IL-4 and IL-5 were determined by qRT-PCR as described above. *P*-values were calculated using the two tailed unpaired Student’s t-test with equal variances. n=3. *, *p*<0.05.

**Supporting Methods**

**Nuclear and cytoplasmic fractionation**

HEK293T cells were plated in 6-well plates (Corning) at a density of 3×10^5^ cells per well and grown to 40-60% confluence overnight. SiRNAs were transfected into cells at a final concentration of 25 nM using Lipofectamine 2000 (Invitrogen) by following the protocol of manufacturer. Fractionation of nuclear and cytoplasmic RNA was performed with the PARIS Kit (Ambion) according to the manufacturer’s instructions at 36 hrs post transfection.

**Quantitative real-time RT–PCR analysis**

The primer sequences were designed as follows:

IL-2-F, 5’-GAACTAAAGGGATCTGAAACAACATTC-3’; IL-2-R, 5’-TGTTGAGATGATGCTTTGACAAAA-3’; IL-4-F, 5’-GCGACTGTGCTCCGGCAGTT-3’; IL-4-R, 5’-TCCAACGTACTCTGGTTGGC-3’; IL-5-F, 5’-TGCAAGGGGGTACTGTGGAA-3’; IL-5-R, 5’-TCTTTGGCTGCAACAAACCAG-3’; β-actin-F, 5’-GCATGGAGTCCTGTGGCA-3’; β-actin-R, 5’-CAGGAGGAGCAATGATCTTGA-3’; ms-IL-2-F, 5’-CTCTGCGGCATGTTCTGGAT-3’; ms-IL-2-R, 5’-ACAACAGTTACTCTGATATTGCTGA-3’; ms-GAPDH-F, 5’-CCAATGTGTCCGTCGTGGATCT-3’; ms-GAPDH-R, 5’-GTTGAAGTCGCAGGAGACAACC-3’; Universal Reverse primer, 5’-GTGCAGGGTCCGAGGT-3’; c-Myc-CEN-RT, 5’-GTCGTATCCAGTGCAGGGTCCGAGGTATTCGCACTGGATACGACCCCTTT-3’; c-Myc-Mip-RT, 5’-GTCGTATCCAGTGCAGGGTCCGAGGTATTCGCACTGGATACGACGACTTT-3’; c-Myc-CEN(Mip)-F, 5’-CGGCGGACCCTCGCATTAT-3’; NPPA-CEN-RT, 5’-GTCGTATCCAGTGCAGGGTCCGAGGTATTCGCACTGGATACGACGGGGCT-3’; NPPA-Mip-RT, 5’-GTCGTATCCAGTGCAGGGTCCGAGGTATTCGCACTGGATACGACAACACT-3’; NPPA-CEN(Mip)-F, 5’-CGGCGGCGCCTCTTTTTAT-3’; IL-6-CEN-RT, 5’-GTCGTATCCAGTGCAGGGTCCGAGGTATTCGCACTGGATACGACAATCTT-3’; IL-6-Mip-RT, 5’-GTCGTATCCAGTGCAGGGTCCGAGGTATTCGCACTGGATACGACAACACT-3’; IL-6-CEN(Mip)-F, 5’-CGGCGGTGGAAACCTTATT-3’; U78-F, 5’-GTGTAATGATGTTGATCAAAT-3’; U78-R, 5’-TTCTTCAGTGTTACCTTTGTC-3’; tRNA-Lys-F, 5’-GCCCGGATAGCTCAGTCGGTA-3’; tRNA-Lys-R, 5’-CGCCCGAACAGGGACTTGAA-3’.
